# Validation of the MSM and NCI Method for Estimating the Usual Intake of Nutrients and Food According to Four Seasons of Seven Consecutive Daily 24 Hour Dietary Recalls in Chinese Adults

**DOI:** 10.3390/nu14030445

**Published:** 2022-01-19

**Authors:** Kun Huang, Dongmei Yu, Qiya Guo, Yuxiang Yang, Xiaoqi Wei, Liyun Zhao, Hongyun Fang

**Affiliations:** Key Laboratory of Trace Element Nutrition of National Health Commission, National Institute for Nutrition and Health, Chinese Center for Disease Control and Prevention, Beijing 100050, China; 15550807252@163.com (K.H.); yudm@ninh.chinacdc.cn (D.Y.); guoqy@ninh.chinacdc.cn (Q.G.); yxyang_ninhccdc@126.com (Y.Y.); xq437073568@163.com (X.W.); fanghy@ninh.chinacdc.cn (H.F.)

**Keywords:** usual intake, NCI, MSM, dietary components, percentage difference, validation, comparison

## Abstract

The Multiple Source Method (MSM) and the National Cancer Institute (NCI) method are used to estimate usual dietary intake from short-term dietary assessment instruments, such as 24 hour dietary recall (24-HRs). However, their performance has not been validated in the Chinese population via nutrition surveys. To validate the accuracy of the MSM and NCI method in estimating usual dietary intake in the Chinese population, 752 individuals from northern and southern China answered four seasons of seven consecutive 24-HRs (one for each season). The true usual dietary intake was considered as the average of the 28 collection days of dietary component intake. Using data sets with consecutive 3 collection days, the usual intakes of the selected dietary components were estimated by MSM, NCI and the within-person mean of three 24-HRs (3 day method). These estimates were compared with the true usual intake at the group and individual level. At the group level, the MSM and NCI method performed similarly, yielding estimates closer to the true usual intake than 3 day method. The percentage differences of the estimates for dietary components not consumed daily from the MSM and NCI method were larger than for the dietary components consumed daily. However, the larger percentage differences were observed in the tail of the usual intake distribution. In general, dietary components with larger variance ratios had greater percentage differences. At the individual level, for overall seasons and dietary components, the biases of individual usual intake did agree for MSM and NCI method, whereas NCI method estimates were closer to true intakes than for the MSM and 3 day method. Similar results were observed in the relative biases of dietary components consumed daily. As with the group level, there was less percentage difference in dietary components consumed daily. Both the MSM and NCI method can be used to estimate usual intake in Chinese populations and are closer to the true usual intake than the traditional mean method, at both group and individual levels.

## 1. Introduction

Usual dietary intake, defined as the average long term dietary intake of an individual, is the exposure of interest when studying the relationship between chronic diseases and diet and is a factor of evaluation when determining the prevalence of inadequate or excessive dietary intakes [1]. There are many dietary assessment instruments such as 24 hour dietary recall (24-HRs), weighed diet records and Food Frequency Questionnaires (FFQ) [2,3,4]. Because of its simplicity and relative accuracy, 24-HRs is a common method for many large nutritional epidemiological surveys and nutritional status surveillances [5,6,7]. The average of a sufficient number of 24-HRs is a similar estimation of usual dietary intake [8]. However, because of the considerable effort and resources involved in conducting frequent visits to households, multiple 24-HRs may be infeasible in large-scale epidemiological studies [9]. Various statistical methods have been proposed to overcome this challenge by using short-term repeated measurements to estimate usual intake (e.g., two 24-HRs) [10,11]. Despite the common principle of considering and removing the within-person variation of intake from the total variation, these methods have different measurement error assumptions, mathematical and statistical methods, implementation complexities, and operating platforms [12].

The Multiple Source Method (MSM) and National Cancer Institute (NCI) method are widely used in many countries to estimate daily intake because they address additional challenges. For instance, they allow the inclusion of covariates in the models to represent the effect of personal characteristics and correlation between probability of consumption and consumption-day amount [13,14]. In addition, there are studies that confirm their validity and discuss limitations [10,12,15]. A simulation study showed that NCI and MSM significantly improved the estimation of the tails of intake distribution when compared to the traditional mean method, and they showed instability when applied to data with large variance ratios or small samples [10]. However, previous studies on the performance of NCI method and MSM have the following problems: first, the simulated data can not completely replace the complex real intake data; second, the Chinese population is not included in the data for validation; third, the data used for validation is mostly collected by two non-consecutive 24-HRs, whereas, the three consecutive 24-HRs is often conducted to collect dietary data in China, such as China Health and Nutrition Survey (CHNS) [16] and China Adult Chronic Disease and Nutrition Surveillance (CACDNS) [17].

In this context, we aimed to validate and compare the NCI method and MSM for estimating the usual intake of food, energy, and nutrients in Chinese adults through four seasons of seven consecutive 24-HRs. To our knowledge, a comparison and validation of methods for usual intake estimation among Chinese populations, for which nutrient composition and dietary variability may differ from European and American populations, has not been previously reported in the literature.

## 2. Materials and Methods

### 2.1. Study Design and Participants

In the study, a representative province from each of the north and south of China was selected. An urban survey site and a rural survey site were selected from each province, and 99 male and 99 female participants were recruited from each survey site. Survey sites were selected based on the level and experience of investigators assigned to the sites, and those who participated in the China Adult Chronic Disease and Nutrition Surveillance in 2015 (CACDNS 2015). Objective sampling was used to recruit participants who were highly compliant and could be surveyed repeatedly. Finally, 780 males and females aged 18–60 years from four sites completed a total of twenty-eight 24-HRs, which were consecutive for 7 days (from Monday to Sunday) each season during December 2019 and December 2020. Twenty-eight participants were excluded for completing less than twenty-three 24-HRs or for reporting implausible energy intakes (outside the range of 600 to 4200 kcal per day for men or 400 to 3500 kcal for women) [18,19].

This study protocol was approved by the Ethics Committee of Chinese Center for Disease Control and Prevention (No. 201519-B), and all participants signed informed consent prior to participating.

### 2.2. Data Collection and Measurements

A uniform set of questionnaires was designed to collect information on participants, including socio-demographic information, health status, diet, and condiments used. The questionnaires were collected by investigators in the household via face-to-face interviews, including consecutive seven 24-HRs at an individual level, and looking for cooking oil and condiments, weighing them at a household level (once a week). When conducting a repeated 24 hour dietary recall in four seasons, the same investigators interviewed the same individuals. Investigators must have gone through the unified training organized by the national project team and have passed the assessment before conducting on-site surveys. 

### 2.3. Dietary Intake Assessment

Participants were asked to recall what they had eaten in the past 24 hours, and all the information about food consumption of each day was recorded, including staple food, side dishes, snacks, fruits, and beverages. The daily food, energy and nutrients intake per person were calculated based on the Chinese Food Composition Tables [20]. In this study, cooking oil and condiment intake were excluded because there was no daily replicate data. The daily consumption of 15 dietary components was calculated, because they present different distributions and scales, and are frequently assessed [21,22,23,24], including energy, macronutrients (carbohydrate, protein and fat), cholesterol, minerals (calcium and iron), vitamin (A, E and B2), and foods (rice, meats, eggs, vegetables, and beans).

### 2.4. Statistical Analysis

The true usual intake of nutrients and foods was defined as the average of more than twenty-three 24-HRs for individuals. This was considered our reference standard for usual intake for further validation and analyses [18]. Usual dietary component intake was estimated by statistical models, using three consecutive 24-HRs, including two weekdays and one weekend, for individuals. However, as each quarter had an eligible consecutive three 24-HRs, the estimation using different collection seasons would be different. To compare the effect caused by the season on the estimation, we estimated the usual intake for each of the four seasons using MSM and NCI method at group and individual levels. For comparison, the 3 day mean of the 24-HRs was calculated for each dietary component. When the participant did not have 3 days of intake, the value of 1 or 2 days was used. 

The NCI method, as its name implies, was proposed by the U.S. National Cancer Institute [13]. It is a two-part mixed-effects regression model in which the first part estimates the probability of consumption using logistic regression with an individual-specific random effect, and the second part uses a mixed-effects linear model to estimate the number of consumption days, which is then transformed to normality using a one-parameter BOX-COX transformation with a person-specific random effect [19]. The first and second parts are linked by allowing two individual-specific effects to be correlated and by including common covariates in both parts of the model. For dietary components that are consumed daily, only the second part of the model is used because the probability of consumption is assumed to be 1. As recommended, we estimated the usual intake at a group level through MIXTRAN and DISTRIB macro version 2.1 and at an individual level through MIXTRAN and INDIVINT macro version 2.1, in SAS software [25]. Moreover, for dietary components with a percentage of zero intake of less than 5%, we chose an amount-model, otherwise we chose a correlation-model or uncorrelation-model, according to whether there was correlation between the probability of consumption and the consumption-day amount [26]. The macro and further details about the NCI method are available at http://riskfactor.cancer.gov/diet/usualintakes/ (accessed on 3 January 2022).

The MSM was developed for use in Europe and is available through an online interface [14]. It is also a two-part model, including probability and consumption amount. However, there are differences in the specific model fitting methods from NCI method. For instance, the second model of MSM is a linear regression model, where the predicted consumption and the corresponding model residuals are estimated with covariates, and then the residuals of the linear regression model are transformed to normality by a two-parameter BOX-COX transformation [27]. Additionally, for the MSM, distributions of the usual intake at a group level are calculated directly from the distributions of the estimated individual usual intake. Therefore, no additional procedures or steps are required to estimate the usual intake at an individual level. The default option of MSM assumes all individuals as habitual consumers in the absence of FFQ data or other external consumption probability information. This setting is equivalent to the assumptions made by the NCI method, so we used this setting to compare MSM and NCI. The MSM was accessed online at http://msm.dife.de/ (accessed on 3 January 2022) to perform the analyses, and further details can be accessed in another publication [14].

Descriptive statistics, including mean, standard deviation, coefficient of variation, percentile distribution, and the percentage of zero intake were calculated for the 3 day, NCI, MSM, and 28 day methods. For NCI method and MSM, we opted for running the models by adjusting for covariates which were the most relevant determinants of dietary intake, including sex, age, and weekend/weekday effect [28]. To determine the Spearman correlation between the probability of consumption and consumption-day amount, we calculated the probability of consumption for 28 days and the mean of consumption-day amount for each dietary component. At the group level, the percentage of difference, defined as the percentage of difference relative to the true usual intake between the estimate and truth, was calculated to compare the estimates of each method. Furthermore, to eliminate seasonal effects, we averaged the percentiles (ranging from 1st to 99th) estimated by three methods in four seasons to express the most expected estimates for this percentile of intake. At an individual level, we calculated bias *MAE* for each method,

(1) $$MAE=\frac{\sum_{i=1}^{N}\left|e_{i}-t_{i}\right|}{N}$$

and the relative percent bias *MAPE*

(2) $$MAPE=\frac{\sum_{i=1}^{N}\left|\frac{e_{i}-t_{i}}{t_{i}}\right|}{N}$$

where $e_{i}$ is the estimated value of each method for the individual i, $t_{i}\;$is the true value of twenty-eight 24-HRs for the individual i, and *N* is the number of individuals in the study.

The software environments employed for estimating usual intake were SAS, to run the NCI method, and Web, to automate the MSM method. The other analyses were performed using SAS version 9.4 (SAS Institute Inc., Cary, NC, USA). All plots were constructed using R version 4.1.2.

## 3. Results

### 3.1. Characteristics of Participants

The mean ± standard deviation age of 752 participants included in the analysis was 39.9 ± 10.2 years and 50.3% were female. Fifty-two-point three percent and fifty-point four percent of the sample were from Southern China and urban areas, respectively. Participants’ education level was categorized as ungraduated from primary school (1.6%), primary or middle school (35.9%), and high school and above (62.5%). The percentages of households with low (<20,000 RMB per year), middle (20,000–50,000 RMB per year), high (>50,000 RMB per year), and unclear income were 1.2, 19.9, 37.1, and 41.8%, respectively. Two-point four percent of participants were lean, whereas 47.6% were overweight or obese. Most participants completed four seasons of a consecutive 7 days of dietary recalls (82.4%) and the percentages of recalls completed from 23 to 27 were 0.9, 0.8, 1.5, 3.5, and 10.9%, respectively. The completion rate of consecutive 3 days of recalls in four seasons was nearly equal (97.1, 97.5, 97.1, and 97.2%).

### 3.2. Characteristics of True Intake (28 Day Mean Intake)

Table 1 presents the descriptive statistics of the true usual intake distributions of 15 dietary components, including energy, protein, fat, CHO, cholesterol, calcium, iron, vitamin A, vitamin E, riboflavin, rice, eggs, beans, vegetables, and meats. The variation of food intake was greater than for energy and most nutrients. The variance ratios for cholesterol, vitamin A, vitamin E, eggs, and beans were all greater than three. For dietary components not consumed daily, correlations between the probability of consumption and the consumption day amount were not observed for all of them, such as for cholesterol, rice, and eggs.

### 3.3. Comparison for Intake at Group Level

**Figure 1:**
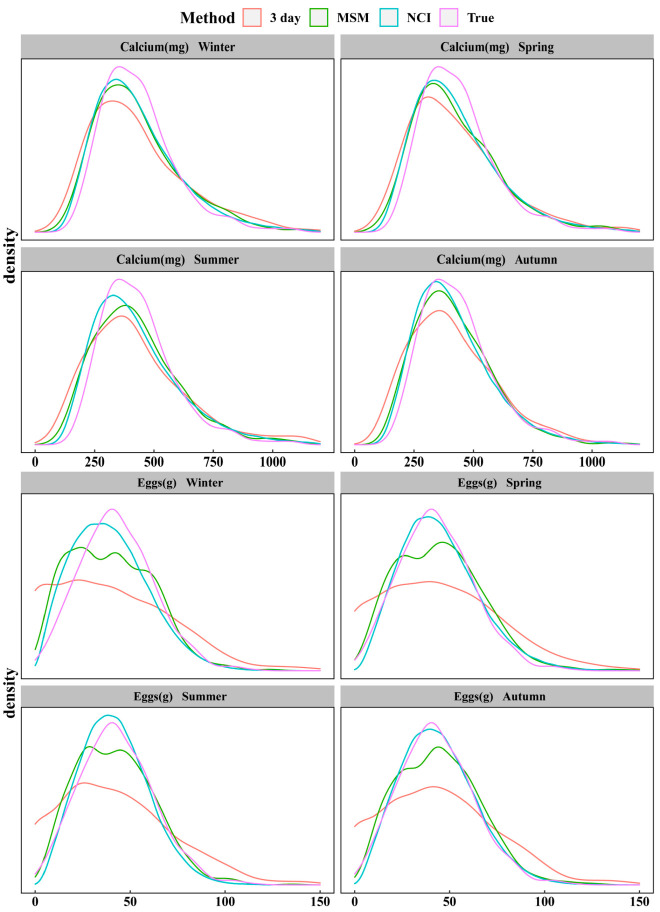
Smoothed distribution curves from the 28 day method, 3 day method, MSM, and NCI based on energy, vitamin A, and eggs. Smoothed distribution curves of all the dietary components evaluated are available in Supplementary Material. 3 day = within-person mean of three 24 hour dietary recalls; MSM = Multiple Source Method; NCI = National Cancer Institute; True = within-person mean of twenty-eight 24-HRs.

Figure 1 shows the distributions based on true intake, estimated from NCI and MSM, and the 3 day method. In most cases, NCI and MSM were better in representing the true intake when compared with the 3 day method. Both NCI and MSM produced similar distributions in four seasons, however, their distributions differed greatly for dietary components not consumed daily. For example, the distribution of eggs usual intake estimated by NCI was closer to the true distribution than for MSM.

Table 2 presents the mean and some percentiles of usual intake distributions for energy, cholesterol, vitamin A, beans, and meats from the NCI method and MSM, as well as the distribution of dietary components from the 3 day average intake and the percentage difference when compared to the corresponding true intake. In general, the 90th percentile of the 3 day method was larger than those of NCI and MSM, while the 10th percentile was smaller than those of NCI and MSM, and the mean and median of the estimated for usual intake agreed for the three methods. The mean and major percentiles of usual intake for selected dietary components did agree for MSM and NCI method, whereas for meats, the 10th percentile of MSM was almost double that of NCI. Most dietary components, except for fat, cholesterol, iron vitamin B2, beans, and vegetables, had seasonal trends, with a decreasing trend from winter to autumn for some components, such as energy, protein, CHO, calcium, vitamin E, rice, and meats, while vitamin A peaked in autumn, beans peaked in summer, and the minimum of cholesterol, vitamin B2, and eggs were in winter.

In most cases, the 3 day method showed a larger percentage difference than the MSM and NCI method for mean and percentiles, especially for the 10th and 90th percentiles. The NCI method and MSM behaved similarly, yielding estimates close to the true value. However, it is clear that both methods were less accurate for the estimation of the 10th and 90th percentiles across most dietary components. Over all seasons and dietary components consumed daily, the ranges of the percentage differences for the mean, median, the 10th, and 90th percentiles of the usual intake distribution estimated by MSM varied from −11.4 to 15.1%, −12.1 to 4.1%, −24.1 to 4.7%, and −18.7 to 12.4%, and the corresponding ranges estimated by the NCI method were from −15.3 to 4.9%, −13.6 to 6.4%, −24.3 to 7.2%, and −14.4 to 11.6%, respectively. For dietary components consumed daily, the percentage difference of mean and percentiles between MSM and NCI was closer, while for dietary components not consumed daily, except for rice, the percentage difference of the 10th percentile estimated by NCI was smaller than for MSM. For example, the MSM estimated the percentage of 10th percentile difference for meats in the range of 57.52 to 119.61%, while NCI estimated this in the range of −27.34 to 19.61%.

**Figure 2:**
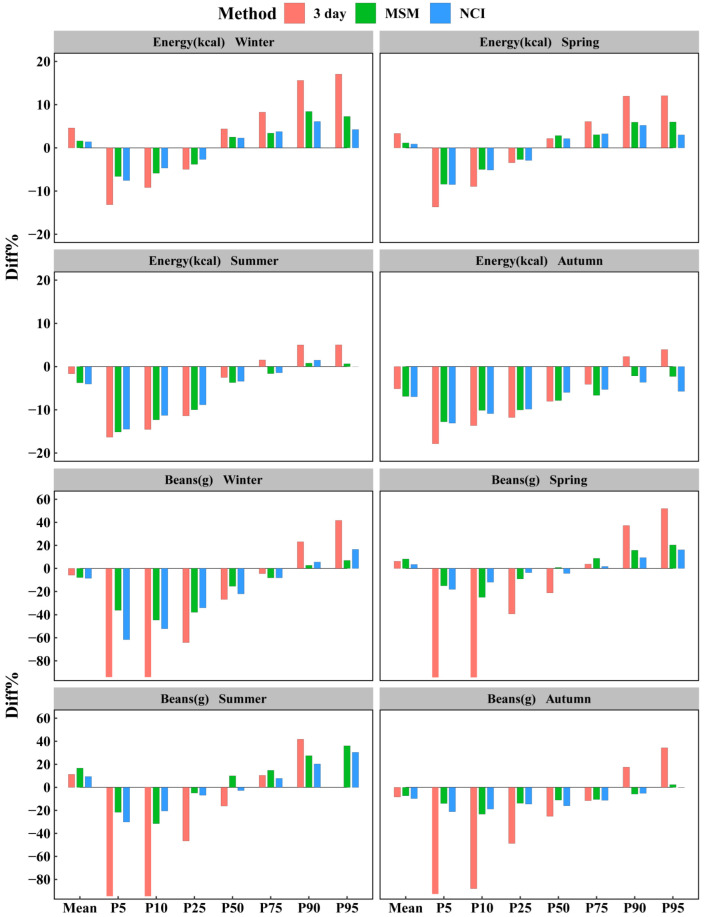
Percentage differences of mean and percentiles estimated from the 3 day method, MSM, and NCI based on energy and beans. Percentage differences of all dietary components evaluated are available in Supplementary Material. The solid line at zero represents no difference. 3 day = within-person mean of three 24 hour dietary recalls; MSM = Multiple Source Method; NCI = National Cancer Institute method; Diff% = percentage differences relative to the 28 day method.

Figure 2 illustrates the bias of mean and percentiles, defined as the percentage difference relative to true intake (the 28 day method), for each of the methods. Except for the mean and median, the 3 day method estimates had a much greater bias than other methods in four seasons. There was no seasonal difference in the bias of MSM and NCI related to vitamins, minerals, and vegetables. For energy and macronutrients, MSM and NCI produced estimates that were close to the truth, with less than a 10% bias for most percentiles in winter and spring. However, for dietary components not consumed daily, there was less bias of percentiles from MSM and NCI in autumn. Both NCI and MSM seemed to shrink the intake distributions more than the 3 day method, resulting in underestimation of the low percentiles and overestimation of the high percentiles. The closer the percentiles were to the median, the less they were underestimated or overestimated. It was worthwhile to note that for certain components such as energy, carbohydrates, rice, and vitamin E in summer and autumn, this result was not appropriate, and their percentiles were always underestimated. 

**Figure 3:**
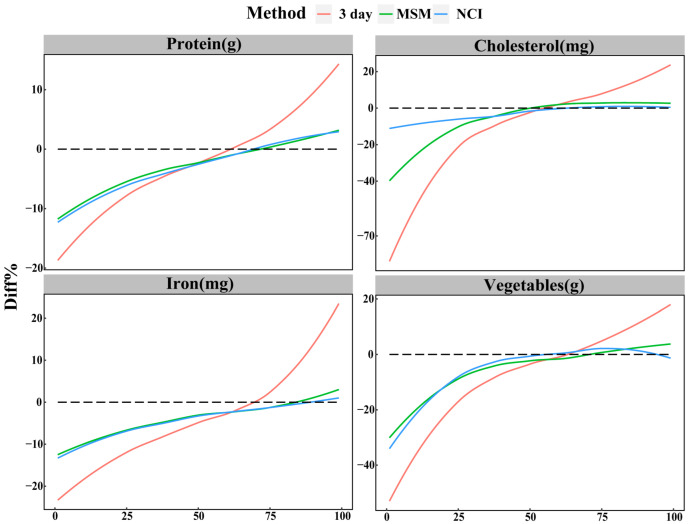
Percentage differences of the percentiles (from 1st to 99th) of estimated from the 3 day method, MSM, and NCI based on protein, cholesterol, iron, and vegetables, after elimination of seasonal effects. The dashed line at zero represents no difference. Percentage differences of all the dietary components evaluated are available in Supplementary Material. 3 day = within-person mean of three 24 hour dietary recalls; MSM = Multiple Source Method; NCI = National Cancer Institute method; Diff% = percentage differences relative to the 28 day method.

To eliminate seasonal effects, we averaged the percentiles (ranging from 1st to 99th) estimated by three methods in four seasons to express the most expected estimate for this percentile of intake. In most cases, the MSM and NCI method behaved similarly, yielding estimates with lower relative biases than the 3 day method. However, for cholesterol and meats, the relative biases above the 25th percentile agreed for MSM and NCI, whereas the relative biases below the 25th percentiles for MSM were twice as high than for the NCI method. As shown in Figure 3, except for cholesterol, the relative biases from the 1st and 99th percentiles of NCI and MSM estimates for protein, iron, and vegetables were generally consistent.

### 3.4. Comparison for Intake at Individual Level

Table 3 shows that the bias estimated by NCI was the lowest for each dietary component in each season, and that of MSM was the second lowest. Additionally, the relative bias of NCI was also the lowest for dietary components consumed daily (except for cholesterol), and MSM was also the second lowest. For beans and meats, however, the relative bias of the 3 day method was lower than for the MSM and NCI method. The relative biases of dietary components consumed daily were smaller than those of dietary components not consumed daily. The relative biases of dietary components with large variance ratios were smaller than those of dietary components with small variance ratios. However, this situation only occurred for dietary components consumed daily. For instance, the variance ratios of vitamin A, vitamin E, and calcium were 4.26, 3.62, and 2.09, respectively, and their relative biases estimated by the NCI method were 25.31~33.82%, 18.6~24.14%, and 19.51~21.79%, respectively.

**Figure 4:**
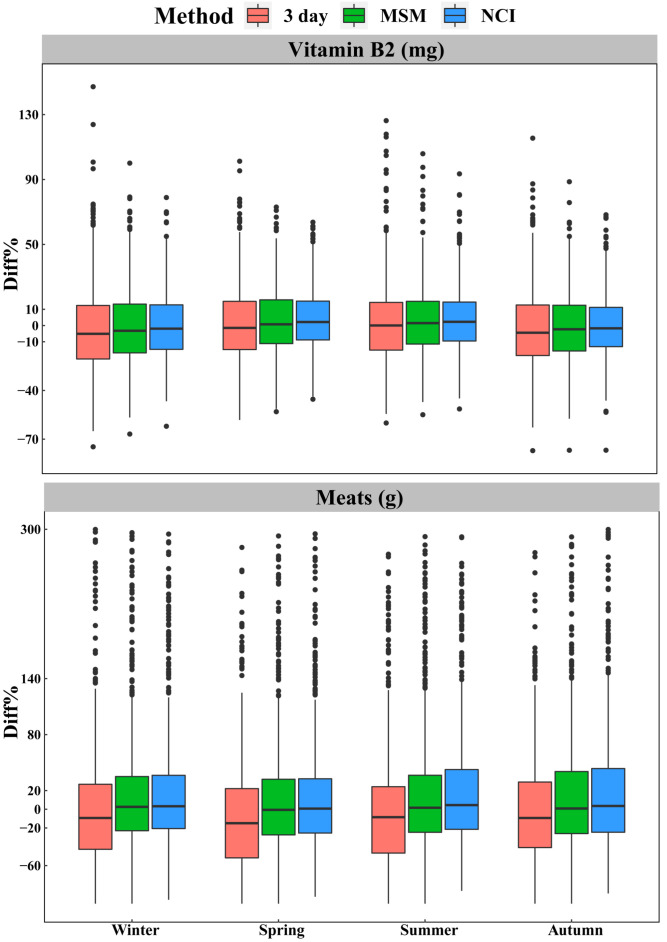
Boxplot of percentage differences estimated by each method based on vitamin B2 and meats for all individuals in four seasons. The percentage difference is the percentage difference error between the estimated value and the true value relative to the true value. Boxplot of all the dietary components evaluated are available in Supplementary Material. 3 day = within-person mean of three 24 hour dietary recalls; MSM = Multiple Source Method; NCI = National Cancer Institute method; Diff% = percentage differences relative to the 28 day method.

Figure 4 presents the precision of NCI for estimating individual intakes, including extreme values; the estimations were better than those of MSM and 3 day method in four seasons, and there was little difference in the precision between seasons. Although the performance of NCI was better, the percentage errors of estimates for vitamin B2 and meats from the MSM and NCI method were almost the same. The percentage differences of the estimates for meats were greater than those of the estimates for vitamin B2. In addition, the usual intakes of individuals with extreme intakes were usually overestimated, especially for dietary components not consumed daily, such as meats. We calculated individuals based on their 28 day average intake and calculated the relative bias for each segment separately, and found that the overestimated individuals were mainly in the segment (below 25th percentile) with low intake, while the relative bias for the other three segments were nearly equal (data not shown).

## 4. Discussion

This study compared and validated two innovative statistical methods and traditional mean method to estimate the usual intake of dietary components at population and individual levels from three consecutive 24-HRs in a sample of adults aged 18 to 60 years living in China. In general, regardless of the levels, the MSM and NCI method provided better estimates of usual dietary intake than the 3 day method. At population level, the usual intake distribution using the MSM and NCI method were similar, except for some situations. However, the NCI method seemed to perform better than the MSM at the individual level.

The results show that the mean and median of intake estimated by each method were approximately the same, which is consistent with the results of previous studies [13,14,29]. This is because the MSM and NCI method were designed to be consistent with estimates using a single 24-HRs per individual. However, when we compared the percentiles at the extremes of estimated intake, the values were significantly different. Similar to previous analyses [29], there was a tendency for estimated intake, based on three methods, to be lower than the true intake at percentiles below the 50th percentile and greater than the true intake for percentiles above the median. There was also a tendency for the difference between estimated intakes, based on the statistical method and the 28 day method, to be larger than the difference between the 3 day method and the 28 day method. However, different results were observed in a study that compared the distribution of usual food intake with twenty 24-HRs: both the MSM and NCI method overestimated the percentiles below the median, particularly up to about the 15th and 20th percentile [30]. We analyzed that the above differences are caused by the differences in the original data, and if the original data are smaller than the true values, then the estimates of the original data corrected by the MSM and NCI method are also underestimated relative to the true values, because both methods compress the tails of the distribution toward the mean. In practice, we are more interested in the magnitude of the error of estimates relative to the true value, rather than the direction of the error. As the difference between the estimated and true values cannot visually reflect the magnitude of the error, we calculated the percentage of difference relative to the true value to compare the performance of methods in different cases. The percentage difference varied with seasons and dietary components, but there was little variation between seasons and more variation between dietary compositions. For example, for many dietary components consumed daily, the percentage differences were within 10%, with higher variation in the lower percentiles. However, for vitamin A and vegetables, the percentage differences were more than 20%, specifically up to about the 5th and 10th percentiles. Similar patterns could be observed among the dietary components not consumed daily. 

Due to different seasons, the performance of the MSM and NCI method to estimate usual intake cannot be directly compared; we instead calculated the mean of the percentiles for four seasons to eliminate the effect of the season. Except for carbohydrates and fats, we found the percentage differences of percentiles from 1st to 99th estimated by MSM and NCI method to be approximately equal for dietary components consumed daily. For carbohydrates, the NCI method had less percentage difference outside the quartiles than for MSM, while the tail percentage difference of the distribution of fat estimated by MSM was less than for that of the NCI method. Nevertheless, the difference between the MSM and NCI method was very subtle, which was not the basis for choosing a certain method. For items not consumed daily, there was a significant difference at the percentiles below 25th percentile between MSM and NCI method, except for the beans and rice. This illustrates that the estimates of the NCI method were closer to the true value than those of MSM. A simulation study has observed that the NCI method provided larger bias in the distribution of usual intake when variance ratio (ratio of within-person variation to between-person variation) was more than nine [10]. 

In this study, however, the variance ratios of items were much less than nine, so we speculated that this difference was related to the choice of model (correlated and uncorrelated models) and the variability of the data. For cholesterol and eggs, the NCI method used an uncorrelated model to estimate usual intake because there was no correlation between the probability of consumption and the consumption-day amount, while MSM used a correlated model to estimate usual intake because it only had one two-part model. The concept of the correlated model is that the probability of consumption of a certain component can affect the amount of that component consumed [13]. In other words, people who eat meats tend to eat larger amounts when they eat them, but only if there is a positive correlation. As the name suggests, the uncorrelated model does not consider the correlation between consumption probabilities and consumption amount [29]. Therefore, the usual intake estimated by MSM for cholesterol and eggs had more extreme higher values than that by the NCI method. Since there were many zero intakes for low consumption probabilities of cholesterol and eggs, the MSM had much larger percentage differences than the NCI method at the low percentiles. When estimating the usual intake of dietary components not consumed daily, hence, it is critical to determine the correlation between the probabilities of consumption and the consumption-day amount. However, there is lack of a standard method to test as to whether to run a correlated or uncorrelated two-part model. Previous studies have included the following methods: First, run correlation model and estimate the Fisher’ s transformation of the correlation coefficient parameter (*p*) and its standard error (which is computed via balanced repeated replication or bootstrap weights) to test the significance of the correlation coefficient between the probability of consumption and the amount consumed [13]; second, it is clear from the available literature that consumers who regularly consume certain dietary components tend to consume more nutrients or foods [31]; third, calculate the proportion of participants who reported consumption on multiple 24-HRs and the median or mean of consumption-day amount. Spearman correlation coefficients between the number of recalls and the daily consumption is calculated [13].

For meats (CV = 89%), the MSM significantly overestimated the low percentiles, resulting in incredible percentage differences, which indicates that the NCI method had better estimates than the MSM for the usual intake of components with high variability. When the distribution of observed intake is extremely skewed, the BOX-COX transformation is not sufficient to convert the observed intake distribution to an approximation normality, which violates a key model assumption and leads to error estimates. Additionally, we found that the degree of improvement of the estimates from the MSM and NCI method relative to the original data was related to the variance ratio. The larger the variance ratio, the greater the improvement because the principle of both methods is to estimate and eliminate the within-person variance from the original data. For this reason, it is more important for dietary components with large variance ratios to correct the original data by the MSM and NCI method to obtain estimates of usual intake that are closer to the true values.

We did not compare the incidence of deficient or excessive foods or nutrients intake among three methods in this study. Since the incidence of deficient or excessive intake was determined by the proportion below the recommended minimum of intake or above the recommended maximum intake, the shape and percentiles of distribution are critical for determining the proportion of a population at risk for inadequacy or excess. Therefore, our results could illustrate that the proportion of below or above recommended consumption was more seriously overestimated or underestimated by using the 3 day method when compared with the MSM and NCI method. Similar results can be observed in previous studies [19,27,29]. Using the simple 3 day method to estimate usual intakes may lead to erroneous conclusions about the severity of public health problems, such as inadequate or excessive intake of certain key nutrients. Using the MSM or NCI method could lead to more appropriate conclusions on dietary status, which could support the development of more accurate public nutrition policies and population-specific nutrition intervention planning.

In general, the NCI method performed better in estimating the usual intake at an individual level, regardless of seasons and dietary components. As can be seen from our analysis, the mean bias of the NCI method, the range of bias, and the bias in the estimation of extreme values were less than those of the MSM and 3 day method. However, there was little apparent difference between the MSM and NCI method. Similar results were observed in a previous study that compared the MSM and NCI method in a large sample of Hispanic/Latino children and adolescents aged 8 to 16 years living in the United States: the usual intakes of individuals estimated by MSM and NCI are close, except when extreme values are estimated [15]. Although the larger percentage differences occurred mainly in individuals with lower intakes, the absolute difference between true values and estimates from the MSM and NCI method was not significant, and we believe this difference may not have a large impact on the evaluation of individual intakes. For dietary components consumed almost daily, including energy, most nutrients, and vegetables, the relative bias was mostly below 30%, and in some cases below 20%, while for cholesterol and foods with low consumption probabilities, the relative bias became more salient. Previous studies have shown that consumption frequency used as a covariate, such as FFQ information, has little effect on results to estimate the usual intake distribution of a population, however, a simulation study demonstrated that the frequency of consumption should be considered a significant predictor when testing the relationship between individual dietary intakes and health outcomes, effectively improving the accuracy of estimates [13,25,27]. In nutritional epidemiology, accurate estimates of each individual’s usual dietary intake are a prerequisite for determining the relationship between dietary intake and disease outcomes, and dietary frequency information is important for accurate estimation. However, additional FFQ information is not always available, in which case the MSM is a better option when compared with NCI method, as it can set up additional settings without this information: First, an external consumption probability value is specified to determine habitual consumption; Second, the MSM assumes that 50% of those not consuming in the short-term measurement are real habitual consumers.

Our study has several strengths. Firstly, this study identifies a gold standard for usual intake, which was obtained from four seasons of seven consecutive 24-HRs in a real population. Secondly, we validated and compared the performance of the MSM and NCI method at the group and individual level. Finally, the validation data for this study were from three consecutive 24-HRs, a commonly used dietary survey method in China, whereas this type of data was not included in previous studies about the comparison and validation of MSM and NCI method.

However, the study still has some limitations. The precision of usual intake estimated by the MSM and NCI method were not compared in this study, so the stability of the performance could not be estimated when the MSM and NCI were applied to different types of data. Additionally, the comparison and validation of both methods for usual intake estimation among child and adolescent population was not conducted in this study, however, their nutrient composition and dietary variability may differ from adults. Finally, because of the lack of FFQ information, we did not evaluate the performance of the NCI and MSM in estimating usual intake for dietary components not consumed daily when the frequency of consumption was included as a covariate in the statistical model.

## 5. Conclusions

In the Chinese adult population, both the MSM and NCI method can provide acceptable estimates of usual intake using a consecutive 3 days of 24-HRs at both the group and individual levels, and their estimates are more representative of usual intake when compared with the traditional within-person mean of three 24-HRs. In general, both NCI and MSM are interchangeable, however, the NCI method was recommended to estimate the usual dietary intake in the following cases: First, for complex survey designs that must include weights, such as CACDNS, the NCI method allows for the inclusion of survey weights; Second, when there is no correlation between the frequency of consumption and consumption-day amount, the more accurate the usual intakes of selected dietary components that are estimated by the NCI method.

## Figures and Tables

**Table 1:** Descriptive statistics of dietary components from twenty-eight 24 hour recalls.

Dietary Components	Means ± SD	CV%	Percentile							VR	Percentage of Zero Intake	r
			5th	10th	25th	50th	75th	90th	95th			
Energy (kcal)	1631.76 ± 468.63	28.72	1028.69	1121.70	1332.30	1562.52	1881.97	2181.79	2450.58	1.35	0.00	-
Protein (g)	68.48 ± 22.07	32.23	40.19	45.21	53.80	64.65	79.36	94.01	105.74	1.43	0.00	-
Fat (g)	38.86 ± 16.87	43.42	15.46	19.35	26.04	37.15	48.69	59.78	69.16	1.98	0.00	-
CHO (g)	252.91 ± 93.94	37.14	140.73	154.93	186.27	231.04	300.47	383.04	431.23	0.92	0.00	-
Cholesterol (g)	383.49 ± 189.64	49.45	117.42	167.05	266.38	363.28	483.74	607.24	687.07	3.09	8.99	0.30
Calcium (mg)	439.03 ± 181.43	41.32	229.35	263.68	323.06	412.32	512.41	639.56	747.78	2.09	0.00	-
Iron (mg)	20.15 ± 6.17	30.63	12.20	13.62	16.01	19.10	23.32	27.51	31.01	2.44	0.00	-
Vitamin A(μgRAE)	424.09 ± 211.53	49.88	198.04	232.12	288.46	378.39	509.77	679.63	774.51	4.26	0.07	-
Vitamin E (mg)	11.99 ± 4.06	33.87	6.75	7.54	9.14	11.41	13.99	17.17	19.39	3.62	0.00	-
Vitamin B2 (mg)	0.83 ± 0.26	31.58	0.49	0.55	0.67	0.81	0.96	1.11	1.24	1.76	0.00	-
Rice (g)	69.18 ± 54.26	78.44	15.49	21.78	34.15	56.47	90.63	125.89	159.97	1.25	26.22	−0.37
Eggs (g)	43.66 ± 21.96	50.29	13.26	18.70	29.63	41.66	55.53	68.28	78.43	3.94	41.82	0.12
Beans (g)	51.37 ± 31.65	61.60	9.21	14.84	27.58	48.01	70.02	92.29	105.86	4.67	30.16	0.61 *
Vegetables (g)	225.69 ± 102.43	45.39	95.64	117.25	151.59	207.51	278.14	349.35	416.25	2.33	3.98	-
Meats (g)	147.44 ± 131.04	88.88	4.18	9.18	37.80	120.87	229.26	313.97	381.25	0.83	26.57	0.89 *

SD—Standard deviation; CV—Coefficient of variation; VR—Variance ratio; Percentage of zero intake—percentage of the number of 24-HRs with zero intake relative to the total number of 24-HRs; *—Spearman correlation between probability of consumption and amount of consumption-day: *p* < 0.05; r—Spearman correlation coefficient.

**Table 2:** The percentiles and mean of the estimated usual intake distributions for selected dietary components from the Multiple Source Method (MSM) and the National Cancer Institute (NCI) method as well as the 3 day average intake.

Dietary Components	Winter				Spring				Summer				Autumn			
	Mean	10th	50th	90th	Mean	10th	50th	90th	Mean	10th	50th	90th	Mean	10th	50th	90th
Energy (kcal)																
3 day	1706.86	1018.78	1631.17	2522.07	1686.00	1021.55	1596.21	2442.64	1604.67	958.71	1523.27	2290.93	1548.06	968.68	1437.18	2232.40
MSM	1657.76	1055.86	1601.42	2365.20	1650.34	1065.66	1606.75	2311.27	1570.69	983.59	1504.74	2198.98	1519.31	1008.09	1439.81	2134.93
NCI	1654.59	1069.43	1598.12	2314.74	1645.94	1064.35	1595.87	2295.62	1566.16	995.06	1509.31	2213.90	1518.06	999.72	1469.31	2102.41
3 day (Diff%)	4.60	−9.18	4.39	15.60	3.32	−8.93	2.16	11.96	−1.66	−14.53	−2.51	5.00	−5.13	−13.64	−8.02	2.32
MSM (Diff%)	1.59	−5.87	2.49	8.41	1.14	−5.00	2.83	5.93	−3.74	−12.31	−3.70	0.79	−6.89	−10.13	−7.85	−2.15
NCI (Diff%)	1.40	−4.66	2.28	6.09	0.87	−5.11	2.13	5.22	−4.02	−11.29	−3.41	1.47	−6.97	−10.87	−5.97	−3.64
Cholesterol (mg)																
3 day	373.25	68.69	340.67	669.87	399.79	100.15	352.06	713.71	385.71	107.96	361.26	683.42	388.63	120.12	352.90	692.27
MSM	362.71	117.18	351.76	612.40	389.47	130.92	360.12	650.03	373.07	141.94	366.08	598.38	378.42	157.21	368.19	615.76
NCI	360.28	147.26	337.57	602.74	386.59	157.20	363.42	644.91	372.53	174.71	358.91	588.87	378.93	179.00	361.82	601.18
3 day (Diff%)	−2.67	−58.88	−6.22	10.31	4.25	−40.05	−3.09	17.53	0.58	−35.37	−0.56	12.55	1.34	−28.09	−2.86	14.00
MSM (Diff%)	−5.42	−29.85	−3.17	0.85	1.56	−21.63	−0.87	7.05	−2.72	−15.03	0.77	−1.46	−1.32	−5.89	1.35	1.40
NCI (Diff%)	−6.05	−11.85	−7.08	−0.74	0.81	−5.90	0.04	6.20	−2.86	4.59	−1.20	−3.02	−1.19	7.15	−0.40	−1.00
Vitamin A (μgRAE)																
3 day	447.06	141.17	353.31	810.93	438.82	151.01	328.13	824.68	373.91	136.50	301.82	672.43	460.76	152.21	360.83	849.18
MSM	426.33	207.37	389.98	692.78	421.00	208.79	365.43	679.43	360.08	176.11	332.64	552.62	441.40	220.58	396.09	713.24
NCI	426.15	211.00	389.22	687.24	416.32	197.08	376.81	684.42	359.03	175.70	326.77	581.95	443.95	213.73	402.01	725.85
3 day (Diff%)	5.42	−39.18	−6.63	19.32	3.47	−34.94	−13.28	21.34	−11.83	−41.19	−20.24	−1.06	8.65	−34.43	−4.64	24.95
MSM (Diff%)	0.53	−10.66	3.06	1.93	−0.73	−10.05	−3.43	−0.03	−15.09	−24.13	−12.09	−18.69	4.08	−4.97	4.68	4.95
NCI (Diff%)	0.49	−9.10	2.86	1.12	−1.83	−15.10	−0.42	0.70	−15.34	−24.31	−13.64	−14.37	4.68	−7.92	6.24	6.80
Beans (g)																
3 day	48.37	0.00	35.19	113.60	54.54	0.00	37.85	126.60	57.16	0.00	40.26	130.80	47.11	1.78	35.97	108.48
MSM	47.31	8.22	40.60	94.77	55.56	11.16	48.40	106.77	59.93	10.17	52.79	117.62	47.64	11.39	42.71	86.99
NCI	46.96	7.10	37.43	97.39	53.12	13.07	45.94	100.93	56.18	11.80	46.69	111.03	46.37	12.05	40.36	87.51
3 day (Diff%)	−5.84	−100.00	−26.70	23.09	6.17	−100.00	−21.16	37.18	11.27	−100.00	−16.14	41.73	−8.29	−88.01	−25.08	17.54
MSM (Diff%)	−7.90	−44.61	−15.43	2.69	8.16	−24.80	0.81	15.69	16.66	−31.47	9.96	27.45	−7.26	−23.25	−11.04	−5.74
NCI (Diff%)	−8.58	−52.16	−22.04	5.53	3.41	−11.93	−4.31	9.36	9.36	−20.49	−2.75	20.31	−9.73	−18.80	−15.93	−5.18
Meats (g)																
3 day	151.11	0.00	111.88	353.17	148.09	0.00	105.33	338.67	147.03	0.00	113.67	336.67	146.64	0.00	103.33	343.75
MSM	148.16	17.76	112.85	320.91	144.19	14.46	111.52	315.66	143.64	15.45	117.38	309.64	142.93	20.16	106.68	325.91
NCI	146.88	9.32	113.22	327.96	143.16	6.67	105.17	327.84	142.19	8.14	115.62	309.90	141.83	10.98	109.88	312.60
3 day (Diff%)	2.49	−100.00	−7.44	12.49	0.44	−100.00	−12.86	7.87	−0.28	−100.00	−5.96	7.23	−0.54	−100.00	−14.51	9.48
MSM (Diff%)	0.49	93.46	−6.64	2.21	−2.20	57.52	−7.74	0.54	−2.58	68.30	−2.89	−1.38	−3.06	119.61	−11.74	3.80
NCI (Diff%)	−0.38	1.53	−6.33	4.46	−2.90	−27.34	−12.99	4.42	−3.56	−11.33	−4.34	−1.30	−3.80	19.61	−9.09	−0.44

3 day—Within-person mean of three 24 hour dietary recalls; MSM—Multiple Source Method; NCI—National Cancer Institute method; CHO—Carbohydrate; RAE—Retinol activity equivalent; Diff%—Percentage difference relative to 28 day method computed for means and percentiles (e.g., (NCI Mean-28 day Mean) × 100/28 day Mean); Percent differences and intakes of all the dietary components evaluated are available in Supplementary Material.

**Table 3:** The bias and relative bias of the usual intake at individual level estimated by the Multiple Source Method (MSM) and National Cancer Institute (NCI) as well as the 3 day average intake for selected dietary components.

Dietary Components	Method	Bias (Relative Bias%)			
		Winter	Spring	Summer	Autumn
Energy (kcal)	3 day	281.29 (17.83)	237.18 (15.03)	239.73 (15.19)	250.72 (15.69)
	MSM	247.13 (15.80)	207.66 (13.30)	211.97 (13.37)	223.59 (13.85)
	NCI	226.33 (14.67)	186.86 (12.18)	191.08 (12.05)	205.09 (12.62)
Protein (g)	3 day	12.84 (19.19)	12.1 (17.42)	11.25 (16.87)	11.72 (17.27)
	MSM	11.38 (17.07)	10.36 (15.26)	9.91 (14.79)	10.58 (15.44)
	NCI	10.56 (15.98)	9.02 (13.70)	9.13 (13.63)	9.83 (14.27)
Fat (g)	3 day	11.98 (32.15)	10.01 (26.70)	10.58 (27.81)	10.48 (27.65)
	MSM	9.73 (27.58)	7.77 (21.23)	8.86 (23.87)	8.82 (23.96)
	NCI	9.00 (27.12)	7.40 (21.58)	7.91 (22.61)	8.11 (23.21)
CHO (g)	3 day	46.86 (19.46)	40.53 (16.89)	40.46 (16.66)	40.19 (16.40)
	MSM	42.87 (17.95)	37.04 (15.67)	37.41 (15.31)	37.56 (15.13)
	NCI	40.04 (17.05)	34.16 (14.87)	34.85 (14.25)	35.71 (14.27)
Cholesterol (mg)	3 day	131.80 (40.89)	129.06 (37.96)	116.56 (37.13)	124.61 (38.72)
	MSM	108.15 (33.38)	105.36 (32.38)	96.31 (31.34)	103.59 (33.85)
	NCI	95.04 (32.31)	89.41 (31.82)	88.75 (32.34)	93.95 (35.49)
Calcium (mg)	3 day	117.25 (27.00)	109.00 (24.54)	108.46 (25.59)	106.39 (24.49)
	MSM	98.69 (23.39)	92.43 (21.43)	92.37 (21.55)	92.72 (21.06)
	NCI	90.19 (21.79)	80.91 (19.51)	83.45 (19.62)	85.67 (19.63)
Iron (mg)	3 day	4.62 (23.03)	4.78 (23.56)	4.30 (21.49)	4.34 (21.37)
	MSM	3.70 (18.78)	3.71 (18.76)	3.42 (17.19)	3.56 (17.52)
	NCI	3.32 (17.17)	3.23 (16.94)	3.02 (15.21)	3.29 (15.88)
Vitamin A (µgRAE)	3 day	187.76 (43.29)	178.35 (40.45)	161.82 (37.93)	183.87 (43.54)
	MSM	135.73 (33.82)	124.80 (30.76)	122.92 (28.39)	135.16 (33.44)
	NCI	120.41 (32.07)	106.36 (27.89)	110.96 (25.31)	121.73 (31.72)
Vitamin E (mg)	3 day	3.98 (33.61)	3.69 (30.49)	3.41 (29.21)	3.39 (28.68)
	MSM	2.99 (26.33)	2.66 (22.71)	2.59 (22.06)	2.45 (20.65)
	NCI	2.63 (24.14)	2.25 (20.22)	2.32 (19.66)	2.23 (18.60)
Vitamin B2 (mg)	3 day	0.17 (21.21)	0.16 (18.70)	0.15 (19.15)	0.16 (18.93)
	MSM	0.15 (18.43)	0.13 (16.31)	0.14 (16.86)	0.14 (16.44)
	NCI	0.14 (16.90)	0.12 (14.76)	0.12 (15.52)	0.12 (14.95)
Rice (g)	3 day	29.13 (54.36)	28.05 (55.88)	23.75 (45.46)	24.67 (45.36)
	MSM	24.64 (47.07)	24.17 (49.88)	20.51 (38.76)	21.34 (38.93)
	NCI	21.58 (47.01)	21.62 (49.15)	19.26 (37.96)	20.54 (38.39)
Eggs (g)	3 day	20.65 (56.49)	20.48 (55.29)	18.38 (51.38)	19.64 (54.38)
	MSM	14.71 (42.36)	14.81 (44.49)	12.97 (40.40)	14.57 (43.93)
	NCI	12.61 (41.31)	12.33 (46.87)	11.87 (44.10)	12.66 (45.80)
Beans (g)	3 day	28.87 (64.04)	30.33 (70.06)	31.49 (69.63)	26.55 (62.17)
	MSM	22.14 (56.64)	23.72 (69.07)	25.17 (70.67)	19.82 (64.16)
	NCI	21.01 (79.31)	22.07 (94.69)	23.70 (96.72)	19.18 (77.23)
Vegetables (g)	3 day	72.38 (34.45)	66.92 (31.31)	66.37 (31.57)	62.65 (29.55)
	MSM	63.67 (30.77)	56.99 (27.05)	58.49 (27.97)	54.54 (26.08)
	NCI	57.31 (30.07)	49.85 (25.60)	52.36 (26.43)	49.24 (24.90)
Meats (g)	3 day	49.37 (64.84)	49.36 (51.34)	46.65 (55.43)	47.69 (53.01)
	MSM	45.55 (114.54)	42.57 (102.01)	43.62 (91.89)	45.18 (117.75)
	NCI	44.63 (105.24)	40.19 (75.48)	43.08 (92.01)	45.14 (91.84)

3 day—Within-person mean of three 24 hour dietary recalls; MSM—Multiple Source Method; NCI—National Cancer Institute Method; Bias—Mean absolute error between estimated and true intake; Relative bias—Mean absolute percentage error between estimated and true intake (relative bias was calculated after excluding individuals with a true intake of zero); CHO—Carbohydrate; RAE—Retinol activity equivalent.

## Data Availability

The data presented in this study are non-public.

## Supplementary Materials

The following supporting information can be downloaded at: https://www.mdpi.com/article/10.3390/nu14030445/s1, Figure S1: Smoothed distribution curves from 28 day method, 3 day method, MSM and NCI based on all dietary components; Table S1: The percentiles and mean of the estimated usual intake distributions for selected dietary components from Multiple Source Method (MSM) and National Cancer Institute (NCI) method as well as the 3 day average intake; Figure S2: Percent differences of mean and percentiles estimated from the 3 day method, MSM and NCI based on all dietary components; Figure S3: Percent differences of the percentiles (from 1st to 99th) of estimated from the 3 day method, MSM and NCI based on all the dietary components after elimination of seasonal effects; Figure S4: Boxplot of percent differences estimated by each method based on all dietary components for all individuals in four seasons. All supplementary tables and figures had all selected dietary components.
