# Supplementary material for: Validation of the MSM and NCI Method for Estimating the Usual Intake of Nutrients and Food According to Four Seasons of Seven Consecutive Daily 24 Hour Dietary Recalls in Chinese Adults
Source: Nutrients. 2022 Jan 19;14(3):445. doi: 10.3390/nu14030445 (PMC8838361; doi:10.3390/nu14030445)
Supplement: Supplementary file 1 [file nutrients-14-00445-s001.zip › Supplementary Materials.pdf]

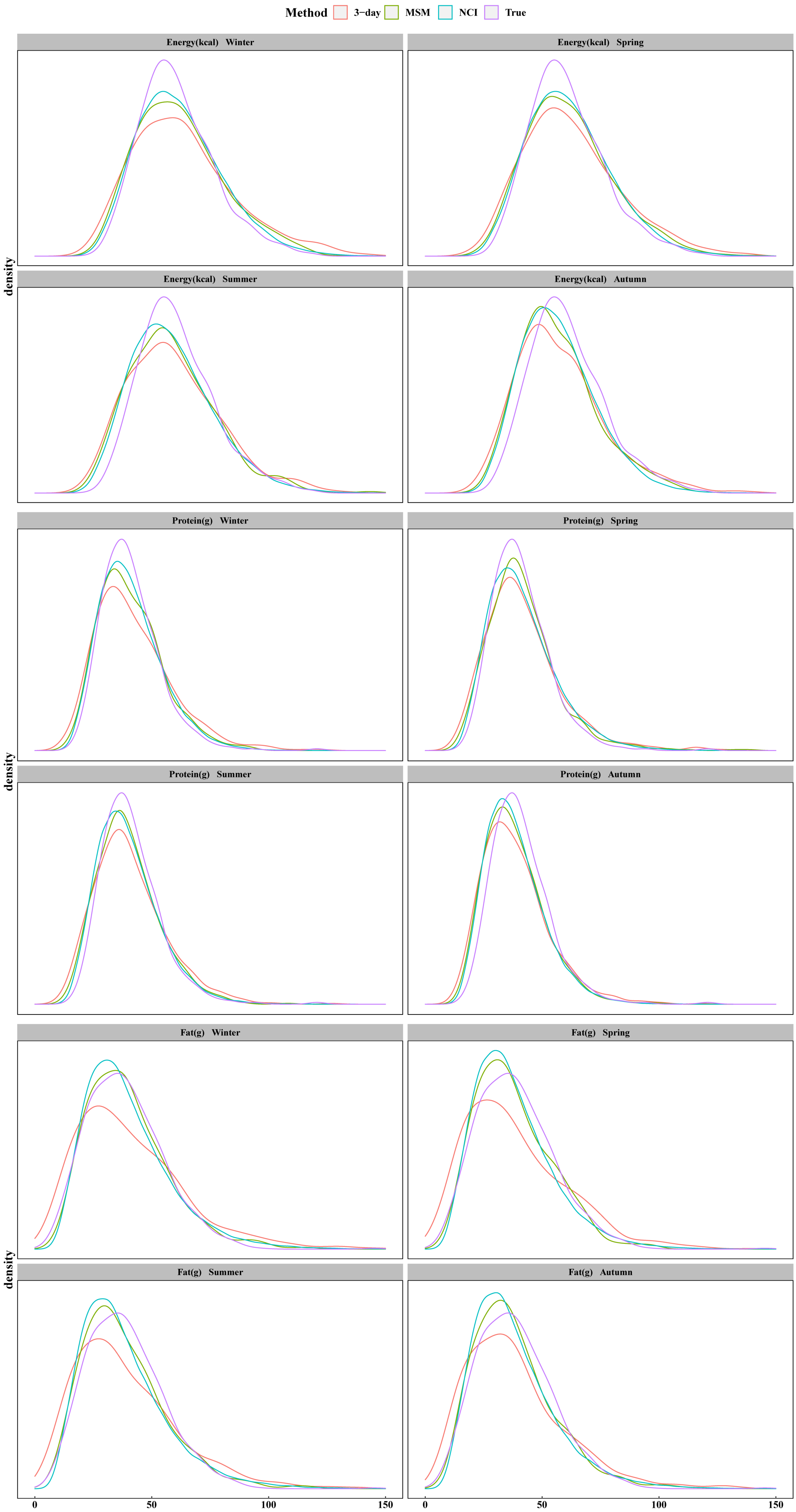

Figure S1. Smoothed distribution curves from 28-day method, 3-day method, MSM and NCI based on all dietary components. 3-day=within-person mean of three 24-hour recalls; MSM= Multiple Source Method; NCI=National Cancer Institute; True= within-person mean of twenty-eight 24-hour recalls.

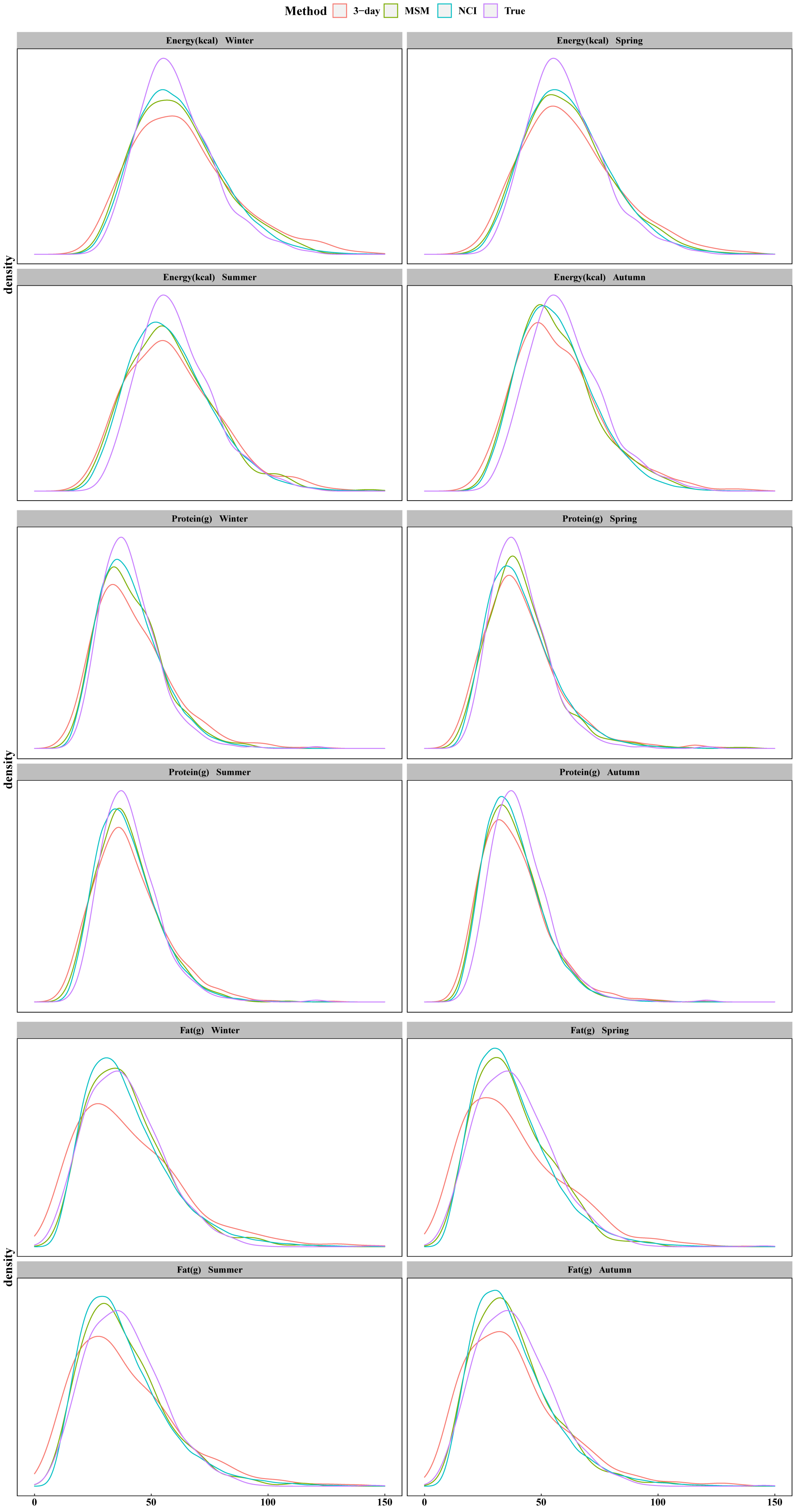

Figure S1. *Cont.*

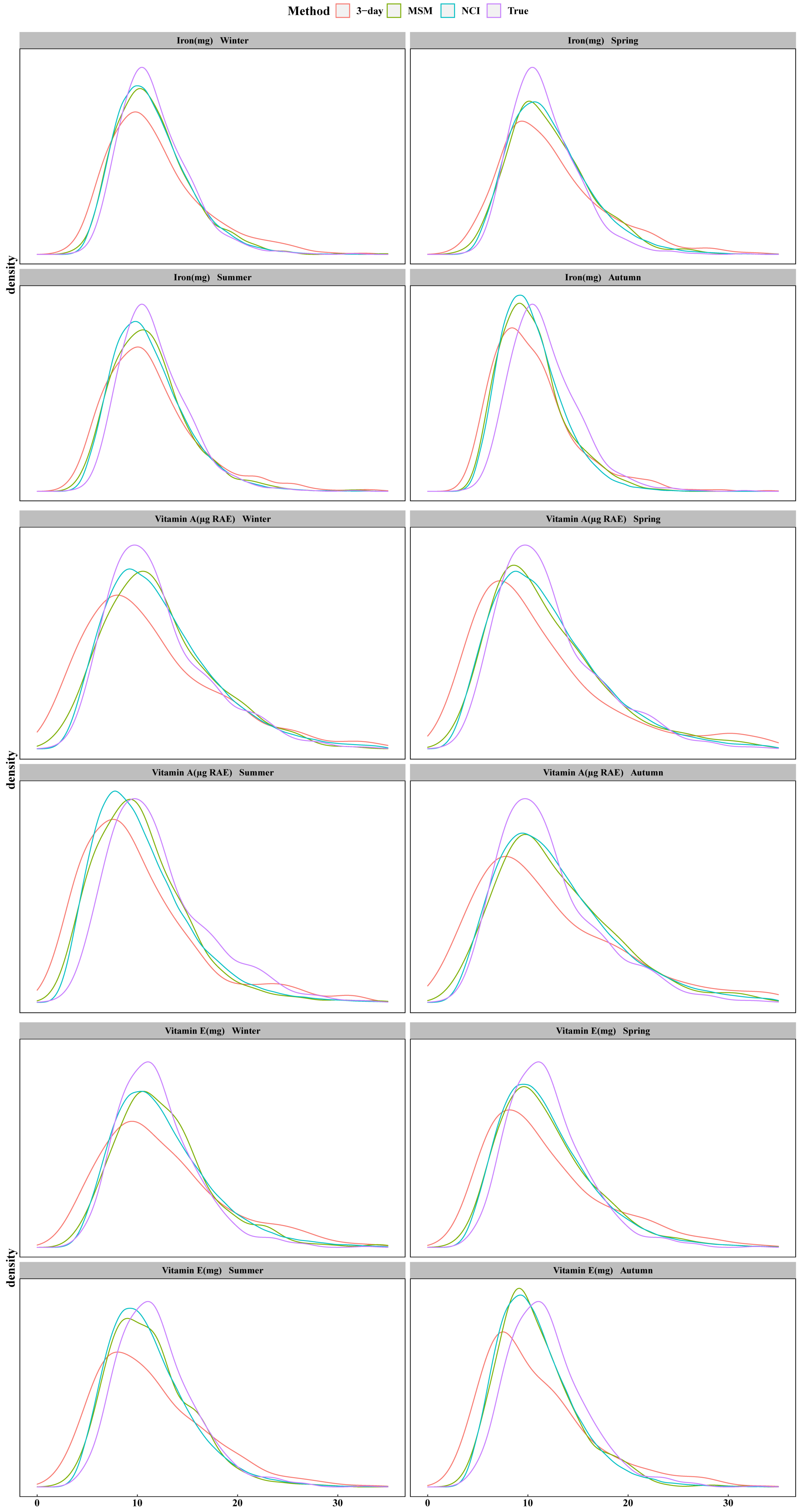

Figure S1. *Cont.*

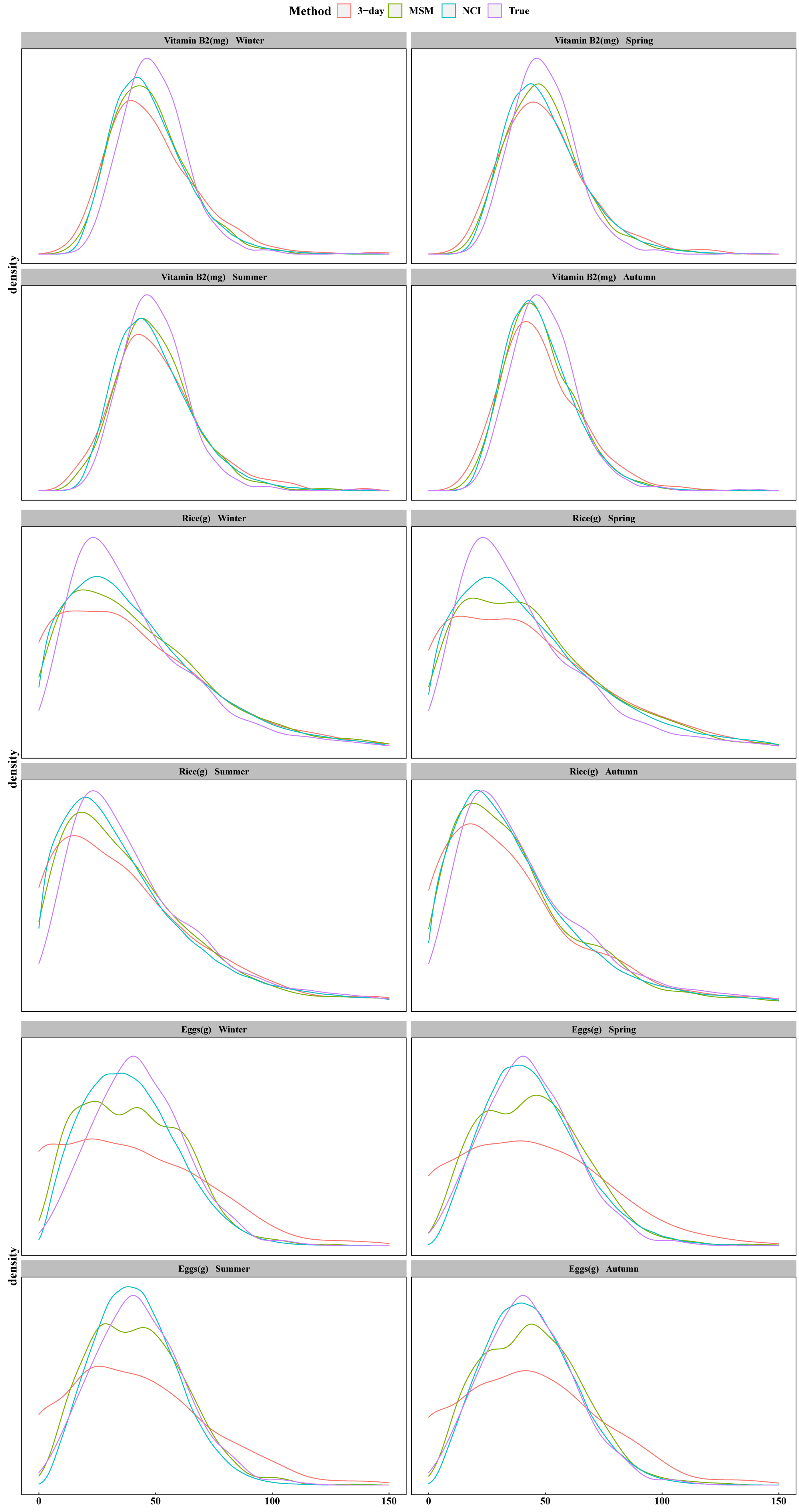

Figure S1. *Cont.*

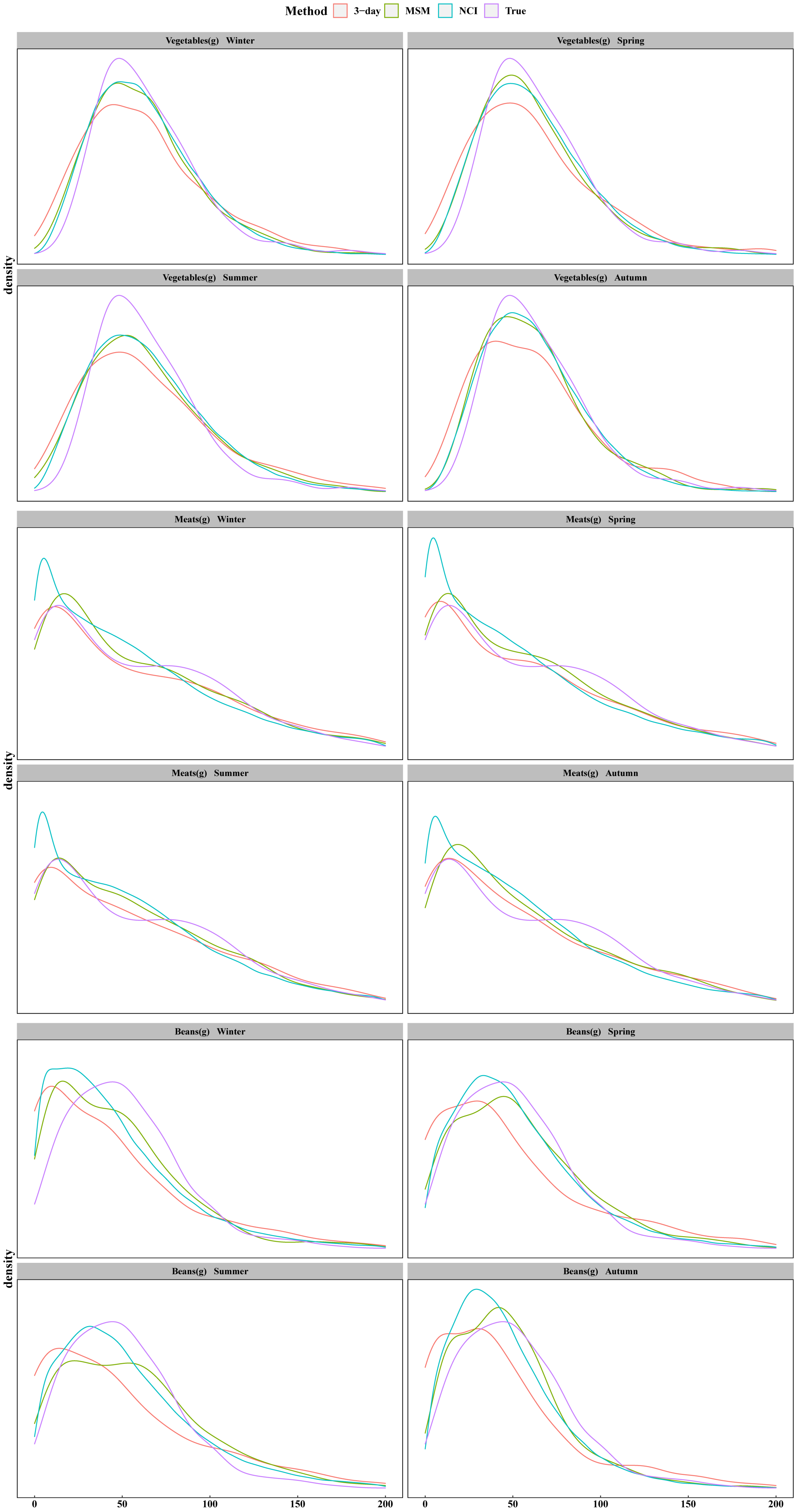

Figure S1. *Cont.*

Table S1. The percentiles and mean of the estimated usual intake distributions for selected dietary components from Multiple Source Method (MSM) and National Cancer Institute (NCI) method as well as the 3-day average intake.

Dietary components	Winter				Spring				Summer				Autumn			
	Mean	10th	50th	90th	Mean	10th	50th	90th	Mean	10th	50th	90th	Mean	10th	50th	90th
Energy(kcal)																
3-day	1706.86	1018.78	1631.17	2522.07	1686.00	1021.55	1596.21	2442.64	1604.67	958.71	1523.27	2290.93	1548.06	968.68	1437.18	2232.40
MSM	1657.76	1055.86	1601.42	2365.20	1650.34	1065.66	1606.75	2311.27	1570.69	983.59	1504.74	2198.98	1519.31	1008.09	1439.81	2134.93
NCI	1654.59	1069.43	1598.12	2314.74	1645.94	1064.35	1595.87	2295.62	1566.16	995.06	1509.31	2213.90	1518.06	999.72	1469.31	2102.41
3-day (Diff%)	4.60	-9.18	4.39	15.60	3.32	-8.93	2.16	11.96	-1.66	-14.53	-2.51	5.00	-5.13	-13.64	-8.02	2.32
MSM(Diff%)	1.59	-5.87	2.49	8.41	1.14	-5.00	2.83	5.93	-3.74	-12.31	-3.70	0.79	-6.89	-10.13	-7.85	-2.15
NCI(Diff%)	1.40	-4.66	2.28	6.09	0.87	-5.11	2.13	5.22	-4.02	-11.29	-3.41	1.47	-6.97	-10.87	-5.97	-3.64
Protein(g)																
3-day	70.32	40.11	65.98	106.34	71.07	39.44	65.10	105.43	68.01	38.28	63.82	102.35	65.08	37.95	60.76	96.68
MSM	68.46	42.10	64.77	98.67	69.66	41.43	65.79	97.97	66.60	40.18	63.87	95.98	63.86	39.75	60.69	92.04
NCI	68.32	42.08	65.16	98.71	69.26	41.67	65.26	101.82	66.48	40.72	63.31	96.34	63.77	39.52	60.73	91.97
3-day(Diff%)	2.69	-11.28	2.06	13.12	3.78	-12.76	0.70	12.15	-0.69	-15.33	-1.28	8.87	-4.96	-16.06	-6.02	2.84
MSM(Diff%)	-0.03	-6.88	0.19	4.96	1.72	-8.36	1.76	4.21	-2.75	-11.13	-1.21	2.10	-6.75	-12.08	-6.13	-2.10
NCI(Diff%)	-0.23	-6.92	0.79	5.00	1.14	-7.83	0.94	8.31	-2.92	-9.93	-2.07	2.48	-6.88	-12.59	-6.06	-2.17
Fat(g)																
3-day	40.87	15.71	36.28	70.37	38.78	15.18	34.03	67.38	39.25	14.95	34.15	69.23	38.70	15.11	34.69	66.99
MSM	39.73	20.25	37.20	61.11	37.75	19.41	34.90	59.68	38.41	18.30	34.77	61.93	37.98	18.36	35.21	61.49
NCI	39.83	19.76	36.25	64.25	37.75	18.93	34.73	60.25	38.30	18.25	34.52	62.80	38.08	18.78	34.66	61.51
3-day(Diff%)	5.17	-18.81	-2.34	17.71	-0.21	-21.55	-8.40	12.71	1.00	-22.74	-8.08	15.81	-0.41	-21.91	-6.62	12.06
MSM(Diff%)	2.24	4.65	0.13	2.22	-2.86	0.31	-6.06	-0.17	-1.16	-5.43	-6.41	3.60	-2.26	-5.12	-5.22	2.86
NCI(Diff%)	2.50	2.12	-2.42	7.48	-2.86	-2.17	-6.51	0.79	-1.44	-5.68	-7.08	5.05	-2.01	-2.95	-6.70	2.89
CHO(g)																
3-day	265.33	139.21	241.58	420.10	264.10	144.53	240.65	418.22	245.68	136.88	223.34	378.66	235.71	132.91	214.31	378.87
MSM	257.46	145.30	236.35	397.51	258.90	150.21	237.73	401.51	240.50	141.74	219.67	366.15	231.41	137.52	212.10	365.14
NCI	256.67	143.97	243.35	386.37	257.88	146.00	245.86	385.09	239.63	135.14	227.39	359.83	230.73	133.66	218.95	342.93
3-day(Diff%)	4.91	-10.15	4.56	9.68	4.42	-6.71	4.16	9.18	-2.86	-11.65	-3.33	-1.14	-6.80	-14.21	-7.24	-1.09
MSM(Diff%)	1.80	-6.22	2.30	3.78	2.37	-3.05	2.90	4.82	-4.91	-8.51	-4.92	-4.41	-8.50	-11.24	-8.20	-4.67
NCI(Diff%)	1.49	-7.07	5.33	0.87	1.97	-5.76	6.41	0.54	-5.25	-12.77	-1.58	-6.06	-8.77	-13.73	-5.23	-10.47
Cholesterol(mg)																
3-day	373.25	68.69	340.67	669.87	399.79	100.15	352.06	713.71	385.71	107.96	361.26	683.42	388.63	120.12	352.90	692.27
MSM	362.71	117.18	351.76	612.40	389.47	130.92	360.12	650.03	373.07	141.94	366.08	598.38	378.42	157.21	368.19	615.76
NCI	360.28	147.26	337.57	602.74	386.59	157.20	363.42	644.91	372.53	174.71	358.91	588.87	378.93	179.00	361.82	601.18
3-day (Diff%)	-2.67	-58.88	-6.22	10.31	4.25	-40.05	-3.09	17.53	0.58	-35.37	-0.56	12.55	1.34	-28.09	-2.86	14.00
MSM(Diff%)	-5.42	-29.85	-3.17	0.85	1.56	-21.63	-0.87	7.05	-2.72	-15.03	0.77	-1.46	-1.32	-5.89	1.35	1.40
NCI(Diff%)	-6.05	-11.85	-7.08	-0.74	0.81	-5.90	0.04	6.20	-2.86	4.59	-1.20	-3.02	-1.19	7.15	-0.40	-1.00
Calcium(mg)																
3-day	449.31	213.72	394.51	737.78	443.09	216.17	393.28	717.87	440.01	193.39	393.31	729.47	425.65	203.18	388.51	671.33
MSM	437.22	236.69	395.39	687.53	435.44	235.15	389.87	676.28	430.98	217.98	402.09	662.00	417.82	230.98	390.95	627.63
NCI	434.48	231.59	398.60	680.52	431.67	228.98	396.68	677.31	431.90	222.88	392.83	687.72	418.24	231.98	389.91	639.55
3-day(Diff%)	2.34	-18.95	-4.32	15.36	0.92	-18.02	-4.62	12.24	0.22	-26.66	-4.61	14.06	-3.05	-22.94	-5.77	4.97
MSM(Diff%)	-0.41	-10.24	-4.11	7.50	-0.82	-10.82	-5.44	5.74	-1.83	-17.33	-2.48	3.51	-4.83	-12.40	-5.18	-1.87
NCI(Diff%)	-1.04	-12.17	-3.33	6.40	-1.68	-13.16	-3.79	5.90	-1.62	-15.47	-4.73	7.53	-4.74	-12.02	-5.44	0.00
Iron(mg)																
3-day	20.51	11.39	18.55	31.58	21.74	12.25	19.33	34.25	19.83	10.80	18.18	30.60	18.30	10.58	16.68	27.67
MSM	19.84	12.62	18.80	28.41	21.16	12.89	19.89	30.92	19.34	12.04	18.46	28.00	17.86	11.49	16.87	25.88
NCI	19.74	12.48	18.79	28.20	21.02	12.95	19.91	30.47	19.22	11.98	18.25	27.70	17.78	11.58	17.02	24.94
3-day(Diff%)	1.79	-16.37	-2.88	14.79	7.89	-10.06	1.20	24.50	-1.59	-20.70	-4.82	11.23	-9.18	-22.32	-12.67	0.58
MSM(Diff%)	-1.54	-7.34	-1.57	3.27	5.01	-5.36	4.14	12.40	-4.02	-11.60	-3.35	1.78	-11.36	-15.64	-11.68	-5.93
NCI(Diff%)	-2.03	-8.37	-1.62	2.51	4.32	-4.92	4.24	10.76	-4.62	-12.04	-4.45	0.69	-11.76	-14.98	-10.89	-9.34
Vitamin A (μgRAE)																
3-day	447.06	141.17	353.31	810.93	438.82	151.01	328.13	824.68	373.91	136.50	301.82	672.43	460.76	152.21	360.83	849.18
MSM	426.33	207.37	389.98	692.78	421.00	208.79	365.43	679.43	360.08	176.11	332.64	552.62	441.40	220.58	396.09	713.24
NCI	426.15	211.00	389.22	687.24	416.32	197.08	376.81	684.42	359.03	175.70	326.77	581.95	443.95	213.73	402.01	725.85
3-day (Diff%)	5.42	-39.18	-6.63	19.32	3.47	-34.94	-13.28	21.34	-11.83	-41.19	-20.24	-1.06	8.65	-34.43	-4.64	24.95
MSM(Diff%)	0.53	-10.66	3.06	1.93	-0.73	-10.05	-3.43	-0.03	-15.09	-24.13	-12.09	-18.69	4.08	-4.97	4.68	4.95
NCI(Diff%)	0.49	-9.10	2.86	1.12	-1.83	-15.10	-0.42	0.70	-15.34	-24.31	-13.64	-14.37	4.68	-7.92	6.24	6.80

Table S1. *Cont.*

Dietary components	Winter				Spring				Summer				Autumn			
	Mean	10th	50th	90th	Mean	10th	50th	90th	Mean	10th	50th	90th	Mean	10th	50th	90th
Vitamin E (mg)																
3-day	13.09	5.74	11.25	22.61	12.35	5.54	10.35	21.60	11.51	5.47	10.33	19.29	11.17	5.33	9.67	18.74
MSM	12.64	7.14	11.85	19.17	12.06	6.77	11.04	18.48	11.27	6.61	10.73	16.67	10.93	6.55	10.12	16.22
NCI	12.58	7.07	11.69	19.17	11.92	6.64	11.05	18.24	11.28	6.50	10.54	16.94	10.90	6.45	10.24	16.15
3-day(Diff%)	9.17	-23.87	-1.40	31.68	3.00	-26.53	-9.29	25.80	-4.00	-27.45	-9.47	12.35	-6.84	-29.31	-15.25	9.14
MSM(Diff%)	5.42	-5.31	3.86	11.65	0.58	-10.21	-3.24	7.63	-6.01	-12.33	-5.96	-2.91	-8.84	-13.13	-11.31	-5.53
NCI(Diff%)	4.92	-6.23	2.45	11.65	-0.58	-11.94	-3.16	6.23	-5.92	-13.79	-7.62	-1.34	-9.09	-14.46	-10.25	-5.94
Vitamin B2 (mg)																
3-day	0.82	0.46	0.76	1.25	0.86	0.47	0.80	1.25	0.85	0.49	0.80	1.27	0.81	0.47	0.76	1.19
MSM	0.80	0.49	0.77	1.13	0.84	0.51	0.80	1.18	0.83	0.51	0.80	1.18	0.80	0.51	0.76	1.13
NCI	0.80	0.48	0.76	1.16	0.83	0.50	0.79	1.22	0.83	0.50	0.79	1.21	0.80	0.50	0.77	1.13
3-day(Diff%)	-1.20	-16.36	-6.17	12.61	3.61	-14.55	-1.23	12.61	2.41	-10.91	-1.23	14.41	-2.41	-14.55	-6.17	7.21
MSM(Diff%)	-3.61	-10.91	-4.94	1.80	1.20	-7.27	-1.23	6.31	0.00	-7.27	-1.23	6.31	-3.61	-7.27	-6.17	1.80
NCI(Diff%)	-3.61	-12.73	-6.17	4.50	0.00	-9.09	-2.47	9.91	0.00	-9.09	-2.47	9.01	-3.61	-9.09	-4.94	1.80
Rice(g)																
3-day	75.19	0.00	58.00	155.90	76.23	5.01	63.55	159.25	63.60	0.00	50.26	131.79	61.83	3.34	49.71	131.70
MSM	73.95	14.66	60.22	146.65	75.55	16.02	66.19	149.15	62.33	12.19	51.06	122.51	61.00	13.46	51.89	124.47
NCI	72.05	14.73	60.36	142.94	74.38	17.14	62.34	145.64	62.12	12.32	50.61	124.96	60.92	12.99	50.67	120.67
3-day(Diff%)	8.69	-100.00	2.71	23.84	10.19	-77.00	12.54	26.50	-8.07	-100.00	-11.00	4.69	-10.62	-84.66	-11.97	4.62
MSM(Diff%)	6.90	-32.69	6.64	16.49	9.21	-26.45	17.21	18.48	-9.90	-44.03	-9.58	-2.68	-11.82	-38.20	-8.11	-1.13
NCI(Diff%)	4.15	-32.37	6.89	13.54	7.51	-21.28	10.39	15.69	-10.21	-43.45	-10.37	-0.74	-11.95	-40.34	-10.26	-4.15
Egg(g)																
3-day	40.85	0.00	36.67	83.33	46.49	0.00	41.17	88.00	43.19	0.00	40.00	85.00	44.55	0.00	41.24	84.62
MSM	39.94	12.34	39.29	67.35	45.45	16.31	43.54	73.68	42.06	16.59	41.06	68.56	44.33	16.81	43.40	71.87
NCI	39.42	14.50	37.59	66.44	44.79	19.72	42.49	72.55	43.74	19.61	42.42	69.11	43.89	19.32	42.34	70.13
3-day(Diff%)	-6.44	-100.00	-11.98	22.04	6.48	-100.00	-1.18	28.88	-1.08	-100.00	-3.98	24.49	2.04	-100.00	-1.01	23.93
MSM(Diff%)	-8.52	-34.01	-5.69	-1.36	4.10	-12.78	4.51	7.91	-3.66	-11.28	-1.44	0.41	1.53	-10.11	4.18	5.26
NCI(Diff%)	-9.70	-22.47	-9.77	-2.69	2.58	5.48	1.99	6.26	0.19	4.84	1.83	1.22	0.53	3.32	1.63	2.71
Beans																
3-day	48.37	0.00	35.19	113.60	54.54	0.00	37.85	126.60	57.16	0.00	40.26	130.80	47.11	1.78	35.97	108.48
MSM	47.31	8.22	40.60	94.77	55.56	11.16	48.40	106.77	59.93	10.17	52.79	117.62	47.64	11.39	42.71	86.99
NCI	46.96	7.10	37.43	97.39	53.12	13.07	45.94	100.93	56.18	11.80	46.69	111.03	46.37	12.05	40.36	87.51
3-day(Diff%)	-5.84	-100.00	-26.70	23.09	6.17	-100.00	-21.16	37.18	11.27	-100.00	-16.14	41.73	-8.29	-88.01	-25.08	17.54
MSM(Diff%)	-7.90	-44.61	-15.43	2.69	8.16	-24.80	0.81	15.69	16.66	-31.47	9.96	27.45	-7.26	-23.25	-11.04	-5.74
NCI(Diff%)	-8.58	-52.16	-22.04	5.53	3.41	-11.93	-4.31	9.36	9.36	-20.49	-2.75	20.31	-9.73	-18.80	-15.93	-5.18
Vegetables(g)																
3-day	224.99	78.33	203.59	400.00	222.65	76.67	194.60	391.67	231.98	77.50	200.17	427.33	221.08	80.67	200.50	390.00
MSM	220.38	95.58	203.02	363.60	219.37	98.17	197.38	358.57	226.75	90.34	203.87	389.29	218.13	101.88	200.34	354.33
NCI	219.44	96.84	205.31	360.55	217.64	92.89	202.75	361.62	227.30	90.44	209.71	386.90	216.96	97.71	203.70	353.44
3-d (Diff%)	-0.31	-33.19	-1.89	14.50	-1.35	-34.61	-6.22	12.11	2.79	-33.90	-3.54	22.32	-2.04	-31.20	-3.38	11.64
MSM(Diff%)	-2.35	-18.48	-2.16	4.08	-2.80	-16.27	-4.88	2.64	0.47	-22.95	-1.75	11.43	-3.35	-13.11	-3.46	1.43
NCI(Diff%)	-2.77	-17.41	-1.06	3.21	-3.57	-20.78	-2.29	3.51	0.71	-22.87	1.06	10.75	-3.87	-16.67	-1.84	1.17
Meats (g)																
3-day	151.11	0.00	111.88	353.17	148.09	0.00	105.33	338.67	147.03	0.00	113.67	336.67	146.64	0.00	103.33	343.75
MSM	148.16	17.76	112.85	320.91	144.19	14.46	111.52	315.66	143.64	15.45	117.38	309.64	142.93	20.16	106.68	325.91
NCI	146.88	9.32	113.22	327.96	143.16	6.67	105.17	327.84	142.19	8.14	115.62	309.90	141.83	10.98	109.88	312.60
3-day(Diff%)	2.49	-100.00	-7.44	12.49	0.44	-100.00	-12.86	7.87	-0.28	-100.00	-5.96	7.23	-0.54	-100.00	-14.51	9.48
MSM(Diff%)	0.49	93.46	-6.64	2.21	-2.20	57.52	-7.74	0.54	-2.58	68.30	-2.89	-1.38	-3.06	119.61	-11.74	3.80
NCI(Diff%)	-0.38	1.53	-6.33	4.46	-2.90	-27.34	-12.99	4.42	-3.56	-11.33	-4.34	-1.30	-3.80	19.61	-9.09	-0.44

3-day—Within-person mean using 3 days; MSM—Multiple Source Method; NCI—National Cancer Institute method; CHO—Carbohydrate; RAE—Retinol activity equivalent; Diff%—Percent difference relative to 28-day method computed for means and percentiles (e.g., NCI Mean−28-day Mean) *100/28-day Mean); Percent differences and intakes of all the dietary components evaluated are available in Supplementary Material.

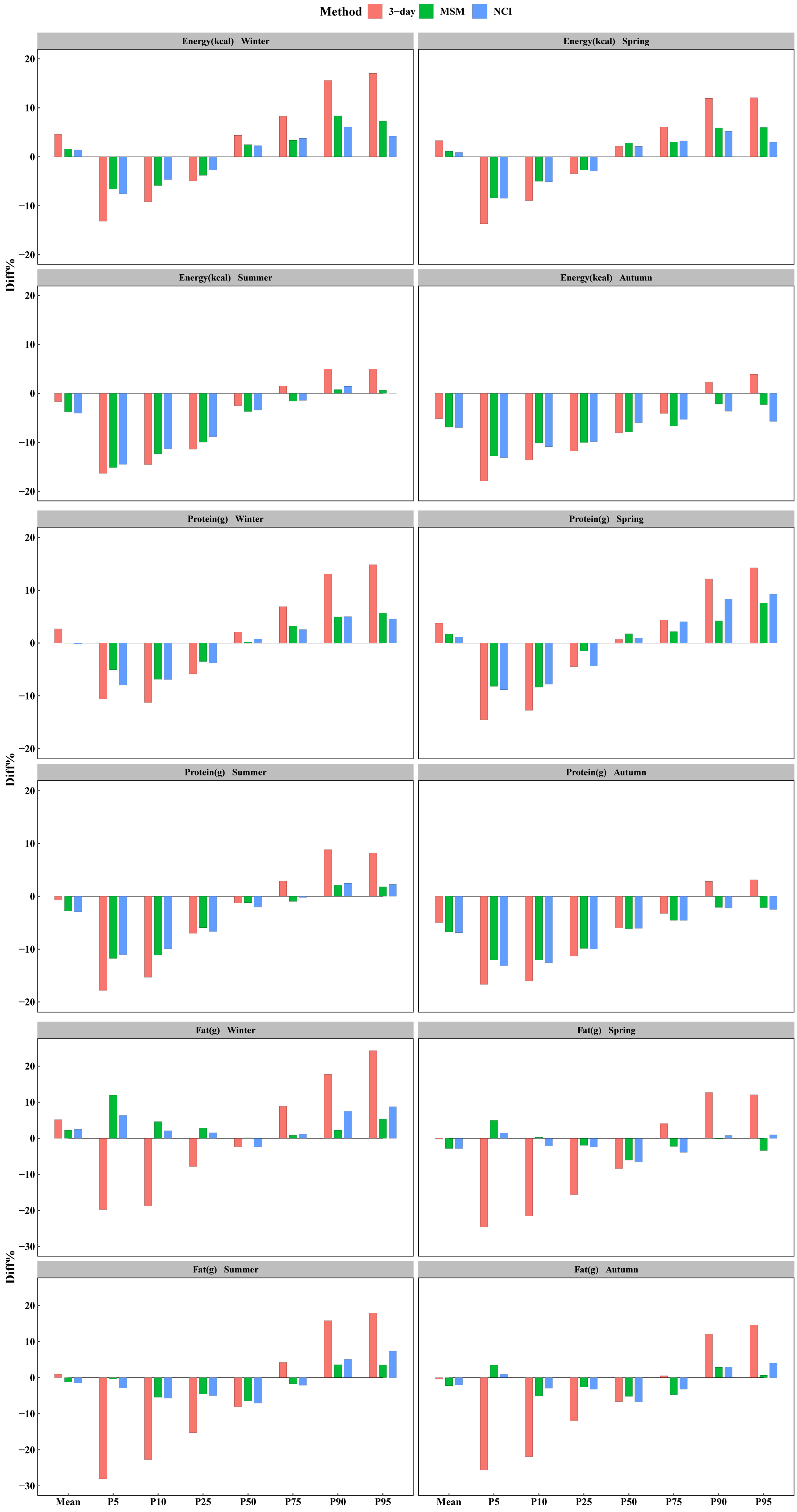

Figure S2. Percent differences of mean and percentiles estimated from the 3-day method, MSM and NCI based on all dietary components. The solid line at zero represents no difference. 3-day=within-person mean of three 24-hour recalls; MSM= Multiple Source Method; NCI=National Cancer Institute method; Diff%=percent differences relative to the 28-day method.

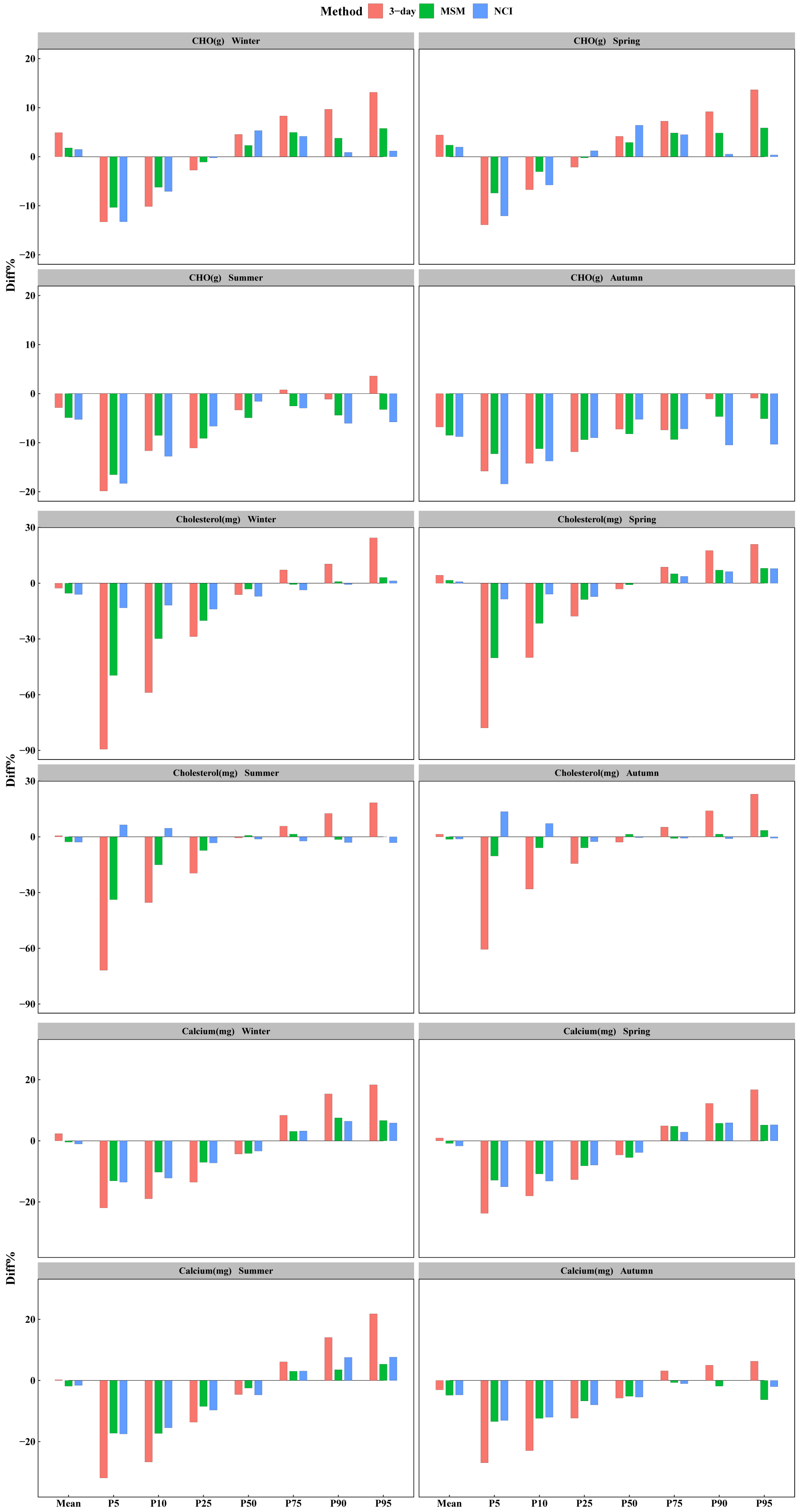

Figure S2. *Cont.*

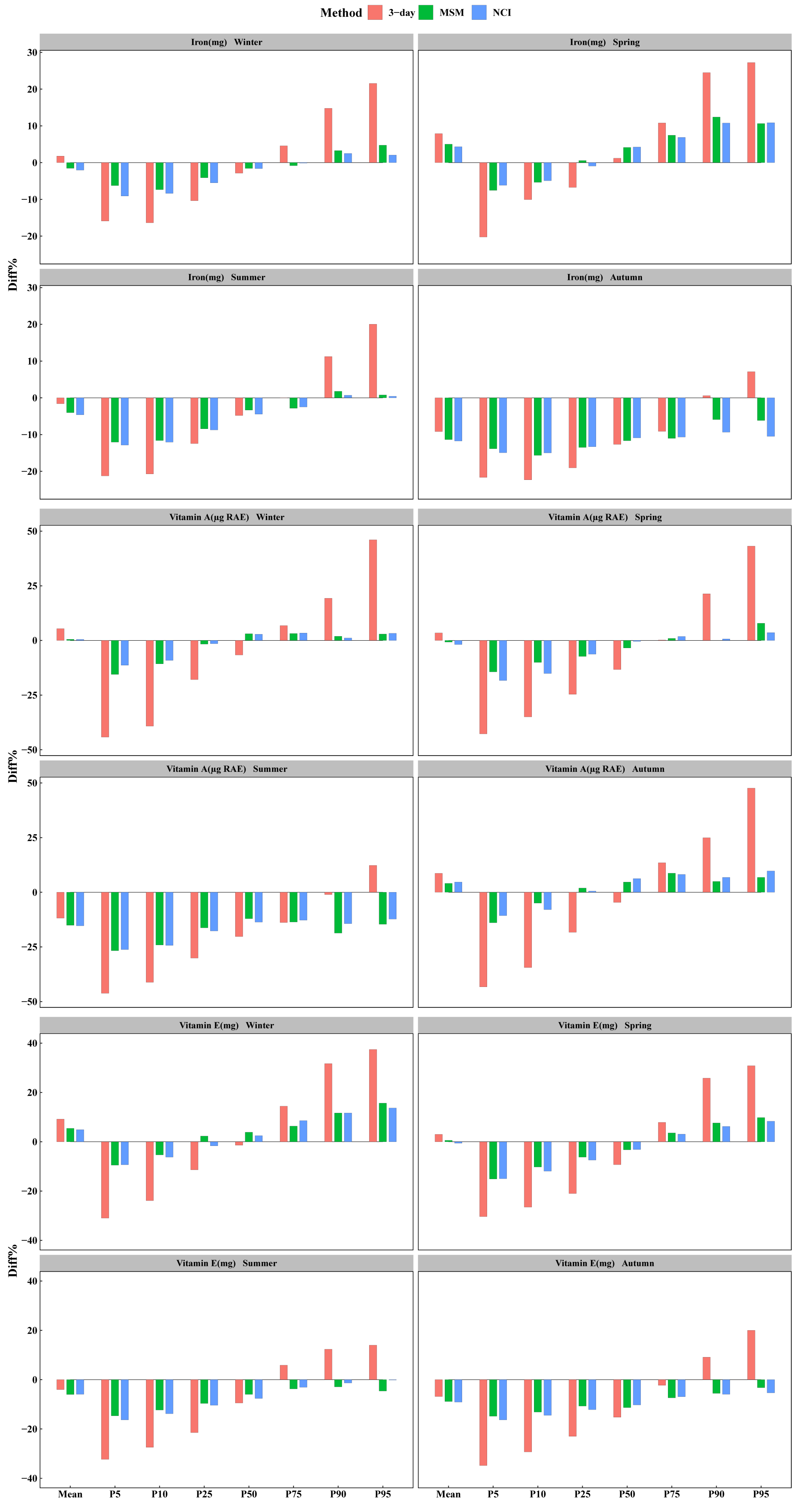

Figure S2. *Cont.*

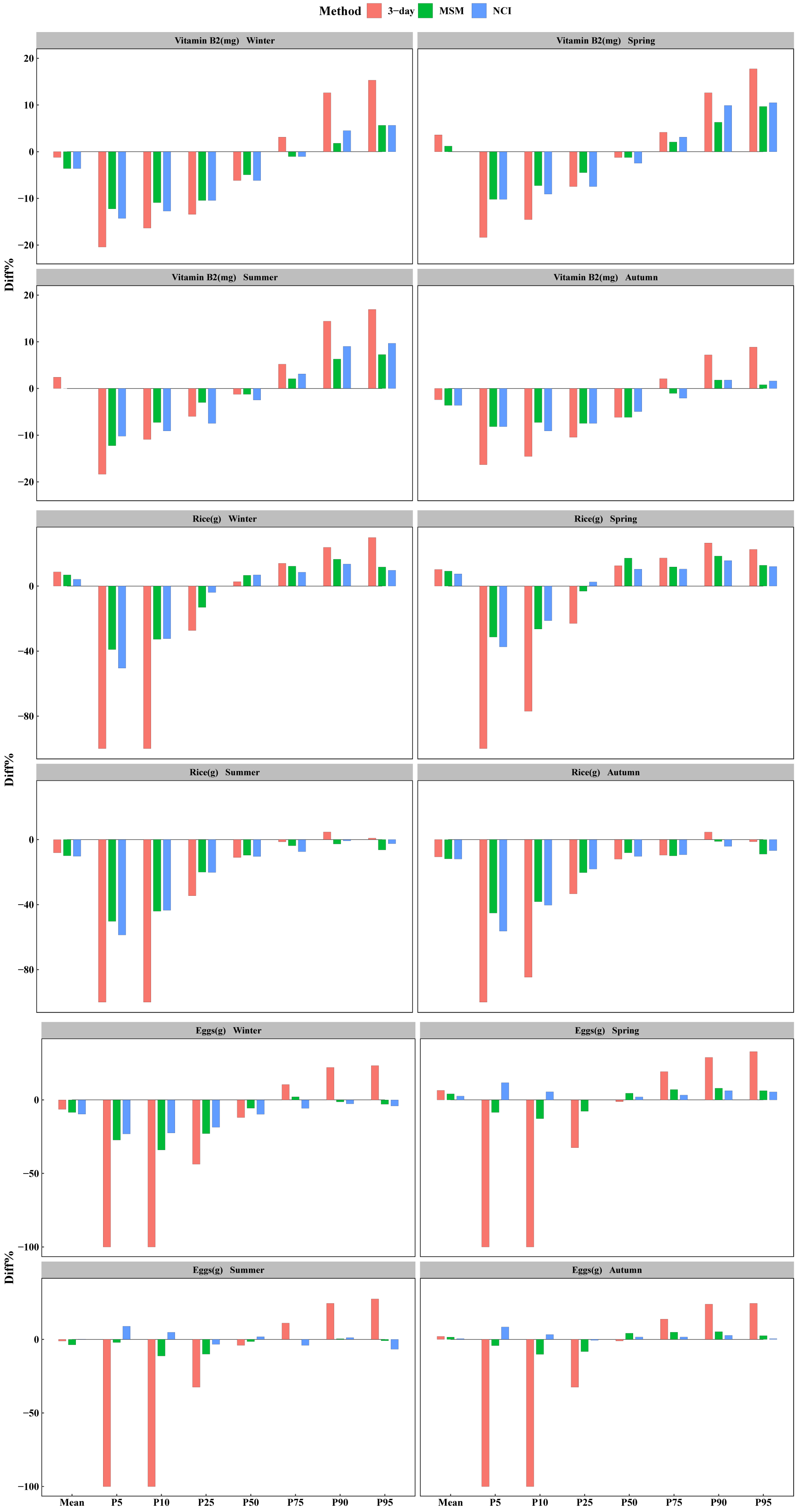

Figure S2. *Cont.*

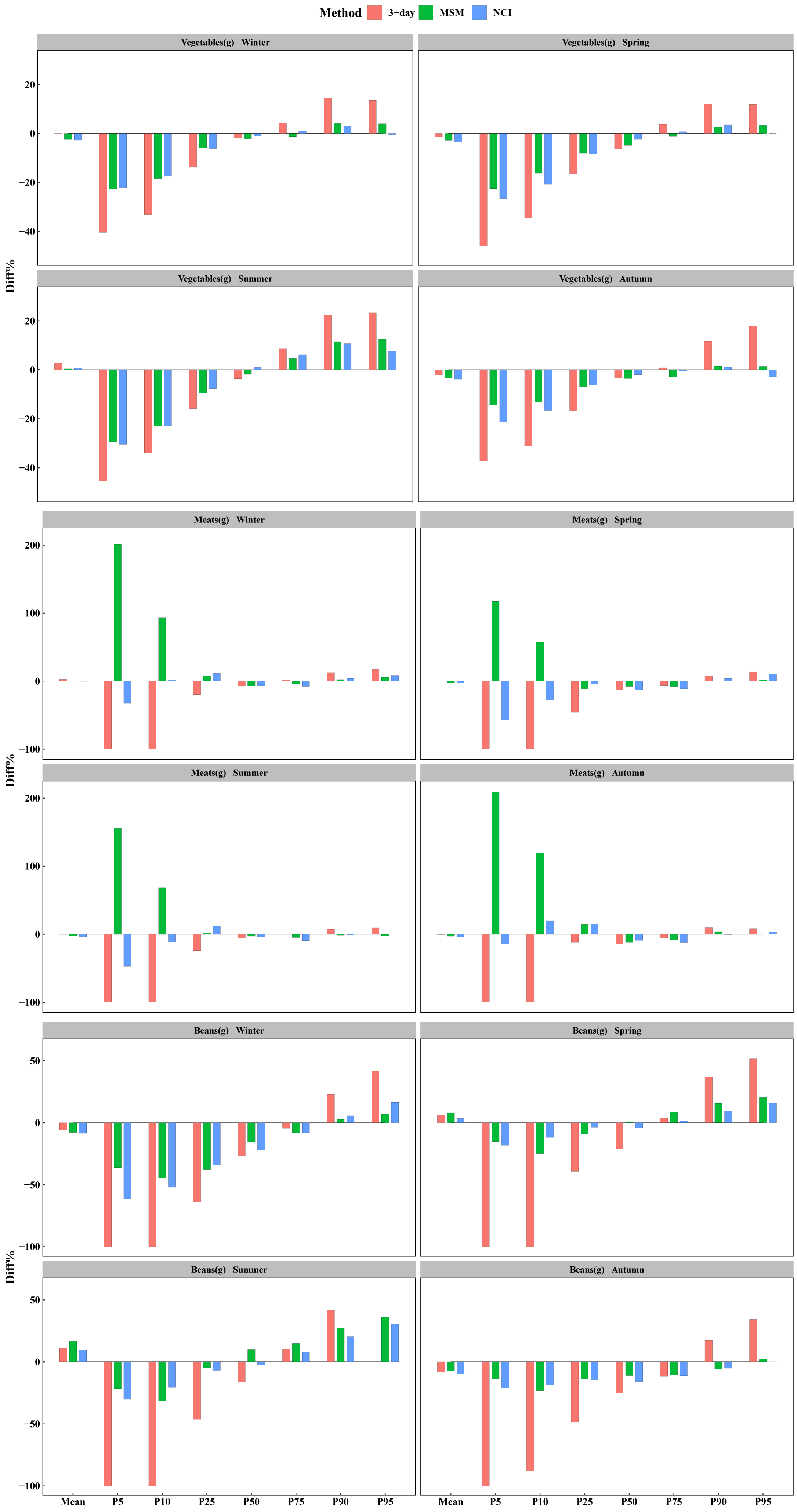

Figure S2. *Cont.*

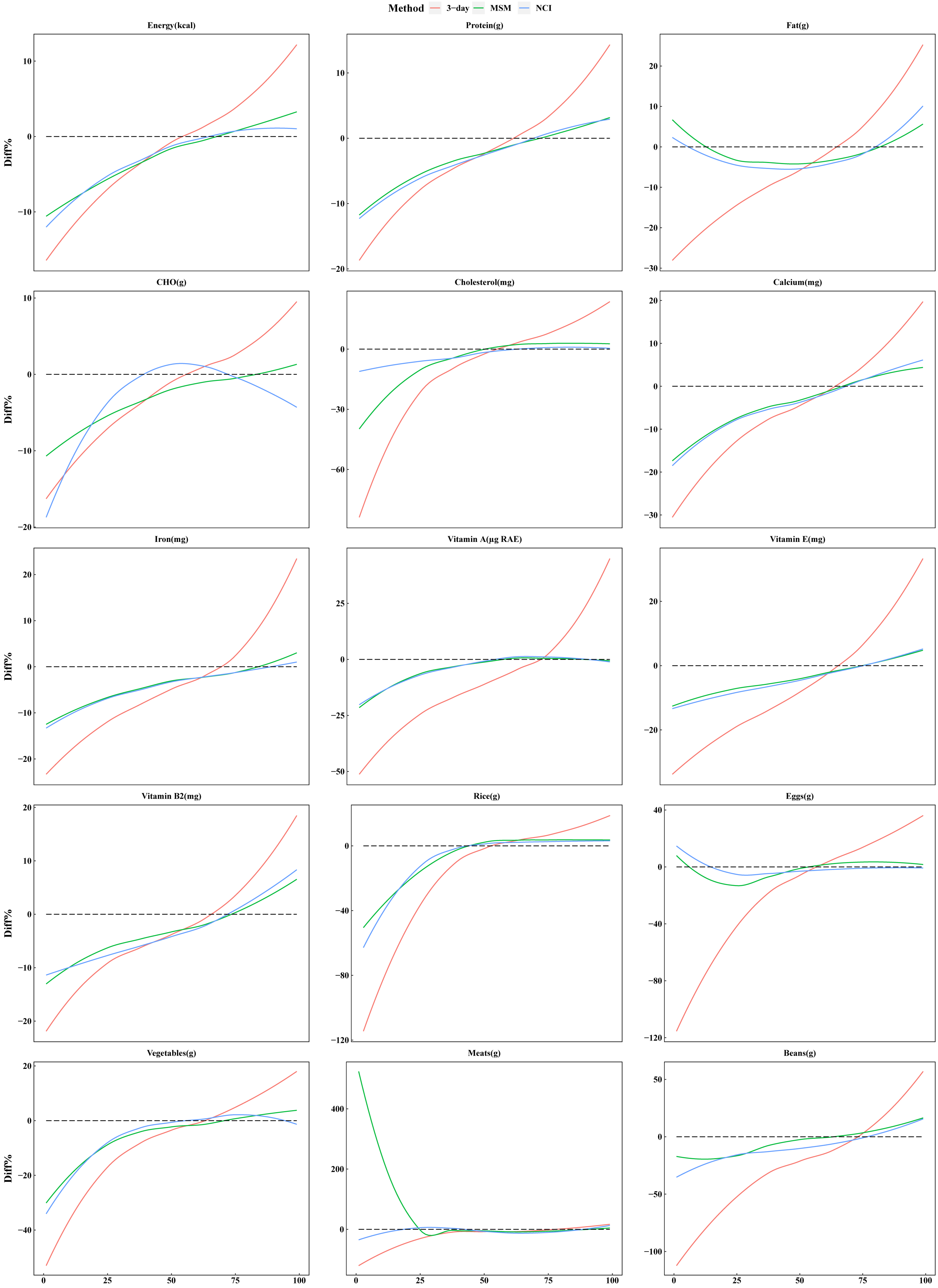

Figure S3. Percent differences of the percentiles (from 1st to 99th) of estimated from the 3-day method, MSM and NCI based on all the dietary components after elimination of seasonal effects. The dashed line at zero represents no difference. 3-day=within-person mean of three 24-hour recalls; MSM= Multiple Source Method; NCI=National Cancer Institute method; Diff%=percent differences relative to the 28-day method.

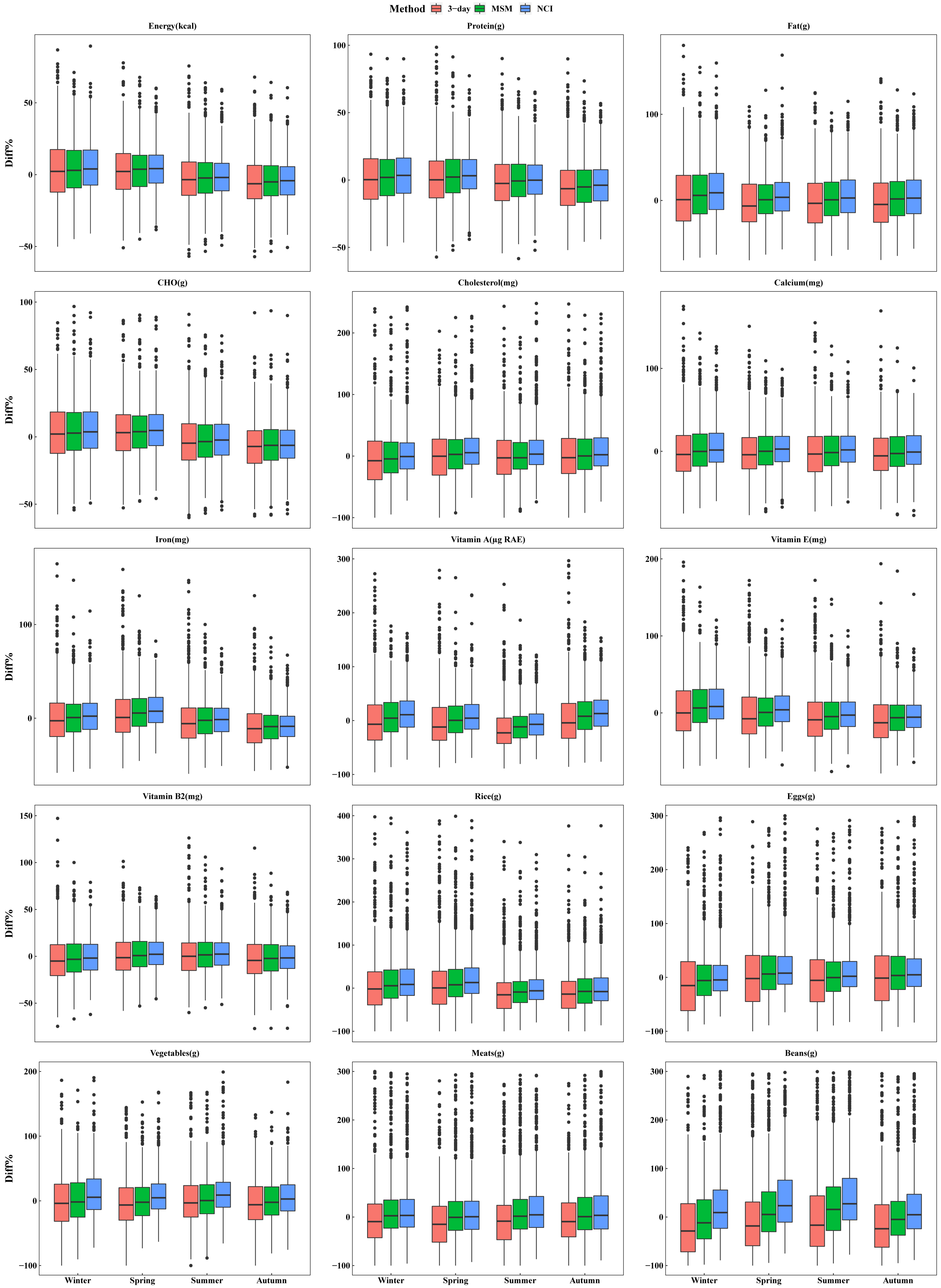

Figure S4. Boxplot of percent differences estimated by each method based on all dietary components for all individuals in four seasons. The percent difference is the percent difference error between the estimated value and the true value relative to the true value. 3-day=within-person mean of three 24-hour recalls; MSM= Multiple Source Method; NCI=National Cancer Institute method; Diff%=percent differences relative to the 28-day method.
